# “Motile-targeting” drug delivery platforms based on micro/nanorobots for tumor therapy

**DOI:** 10.3389/fbioe.2022.1002171

**Published:** 2022-09-16

**Authors:** Di Zhang, Shuyi Liu, Jianguo Guan, Fangzhi Mou

**Affiliations:** State Key Laboratory of Advanced Technology for Materials Synthesis and Processing, Wuhan University of Technology, Wuhan, China

**Keywords:** micro/nanorobots, micro/nanomotors, drug delivery, tumor targeting, self-propulsion

## Abstract

Traditional drug delivery systems opened the gate for tumor-targeted therapy, but they generally took advantage of enhanced permeability and retention or ligand-receptor mediated interaction, and thus suffered from limited recognition range (<0.5 nm) and low targeting efficiency (0.7%, median). Alternatively, micro/nanorobots (MNRs) may act as emerging “motile-targeting” drug delivery platforms to deliver therapeutic payloads, thereby making a giant step toward effective and safe cancer treatment due to their autonomous movement and navigation in biological media. This review focuses on the most recent developments of MNRs in “motile-targeting” drug delivery. After a brief introduction to traditional tumor-targeted drug delivery strategies and various MNRs, the representative applications of MNRs in “motile-targeting” drug delivery are systematically streamlined in terms of the propelling mechanisms. Following a discussion of the current challenges of each type of MNR in biomedical applications, as well as future prospects, several promising designs for MNRs that could benefit in “motile-targeting” drug delivery are proposed. This work is expected to attract and motivate researchers from different communities to advance the creation and practical application of the “motile-targeting” drug delivery platforms.

## 1 Introduction

Tumors, especially cancer, a disease that has plagued humans for centuries, are ranked as a leading cause of death. It is estimated that 19.3 million new cancer cases and nearly 10.0 million cancer deaths occur worldwide every year (Sung et al., 2021). Surgery, chemotherapy, and radiotherapy are the three main ways to treat cancer. Chemotherapy is the most popular one, which uses powerful drugs to stop or slow the growth of cancer cells. However, traditional cancer chemotherapy not only has side effects but also suffers from low effectiveness as cancer can display adaptive evolution towards some therapeutic agents (Ibragimova et al., 2017). As a result, researchers strive to find effective ways to directly deliver chemotherapeutic agents to tumor sites by developing various passively and actively targeted delivery systems based on nanorods (Ju et al., 2014), liposomes (Camp et al., 2013; Senzer et al., 2013), polymeric micelles (Torchilin, 2002), dendrimers (Guo et al., 2012; Gao et al., 2015), nanotubes (Liu et al., 2008), self-assembling peptides (Liu et al., 2007; Wang W et al., 2019), and so on. Even though there is a wide variety of drug delivery systems, the passive targeting drug delivery system relying on enhanced permeability and retention (EPR) often has severe side effects due to the low drug accumulation near the targeting area. An active targeting delivery system that mainly depends on ligand-receptor mediated interaction is limited by the recognition range (<0.5 nm) (Bae and Park, 2011; Dehaini et al., 2016). In addition, the targeted drug delivery may be hindered by the abnormal and complex structure of the vasculature and interstitial hypertension (Jain and Stylianopoulos, 2010). Despite their inadequacy to cure cancer, the massive research on multiple nanomaterials, fabrication methods, and drug delivery processes has paved the way for the new generation of drug carriers.

**FIGURE 1:**
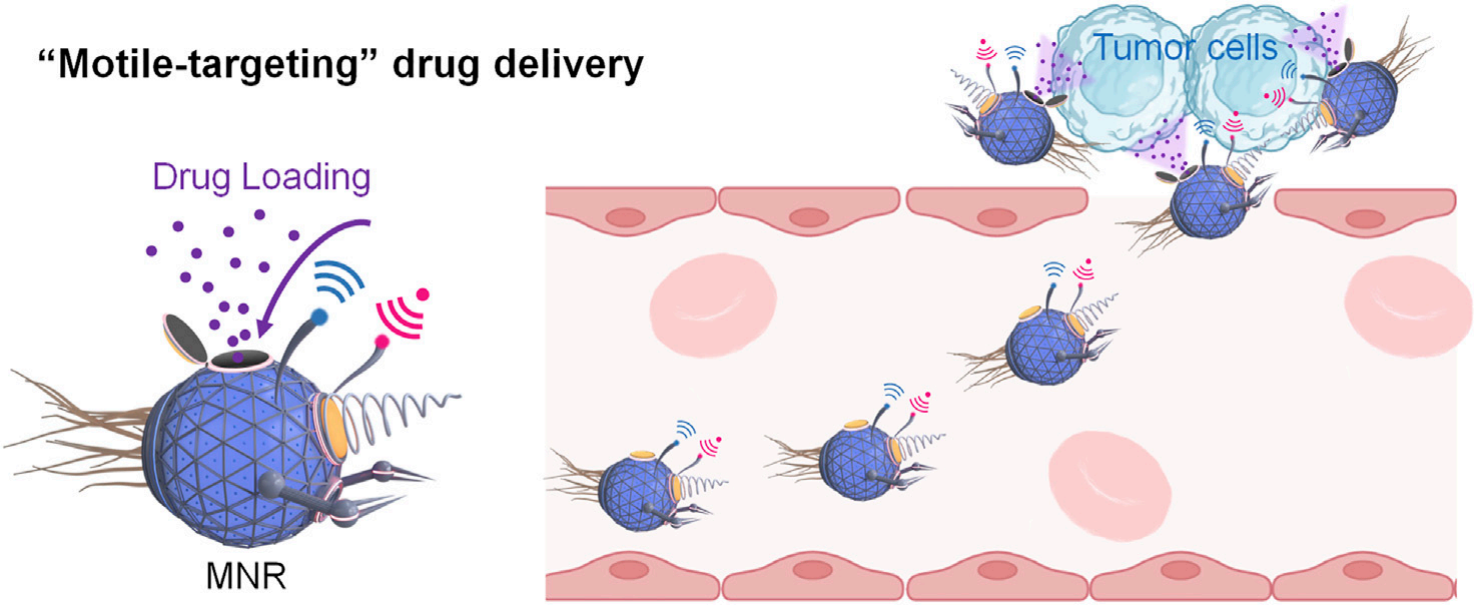
Schematic illustration of “motile-targeting” drug delivery of micro/nanorobots (MNRs).

To achieve effective and precise cancer treatment, drug carriers should be distributed at a reasonable time and space near the target sites. Thus, the loaded drugs can be released and taken in by the cells. Therefore, ideal drug carriers must comply with the capabilities of avoiding obstacles, escaping from the immune system, and displaying controllable motion. Emerging Micro/nanorobots (MNRs, or micro/nanomotors, microswimmers, active colloids) are potent machines in the micro/nanoscale that can transform diverse energy forms into mechanical movement. In general, they can be classified into three types: chemically-powered MNRs, external-field-powered MNRs, and biohybrid MNRs. They can move at a controlled speed and direction by taking advantage of inherent chemotaxis, external-field navigation, or precisely designed structures. And they can cross many biological barriers, such as blood-tumor barriers and dense extracellular matrixes (Lin et al., 2021). Therefore, the MNRs can deliver force, energy, and cargoes to target sites and perform various biomedical operations, including photothermal therapy (Wu et al., 2014), bioimaging (Aziz et al., 2020), photodynamic therapy (Gao et al., 2019), cartilage repair (Go et al., 2020), detoxification (Zhu et al., 2015), microbiopsy (Xi et al., 2013), and so on. The MNRs are of increasing interest in tumor therapy, as they can deliver therapeutic agents to specific tumor sites with high precision in a “motile-targeting” manner (in contrast to passive and active drug delivery of non-motile carriers), thereby reducing the required dose of administrated anti-tumor drugs and their systemic side effects and increasing the treatment efficacy (Figure 1). For instance, it was found that motile nanocarriers driven by magnetic fields could deliver various hydrophobic drugs into the deep tumor tissue, and showed improved tumor treatment efficacy by 20% *in vivo* compared with ordinary passive nanocarriers (Zhu et al., 2021).

This review focuses on the state-of-the-art “motile-targeting” drug delivery of MNRs. At first, traditional tumor-targeted drug delivery strategies and MNRs with different propelling mechanisms are briefly introduced. Then, representative applications of MNRs in “motile-targeting” drug delivery are systematically summarized. After that, current challenges and future outlook toward *in-vivo* applications of each type of MNR have been discussed, and several promising design strategies for MNRs that may facilitate their “motile-targeting” drug delivery have been proposed. This work is expected to attract and motivate researchers from nanomedicine, MNRs, and other fields to advance the creation and practical application of the “motile-targeting” drug delivery platforms.

## 2 Targeting drug delivery systems for tumor therapy

### 2.1 Passively targeted drug delivery systems

Passively targeted drug delivery systems (PTDDSs) for tumor therapy mainly relies on the EPR effect that largely depends on the unique pathophysiological characteristics of tumors and characteristics of nanomaterials, and the blood circulation parameters (e.g., circulation time, phagocytosis, etc.) (Alexis et al., 2010; Dai et al., 2016; Kumari et al., 2016). Researchers develop a diversity of passive drug carriers according to the physiological and nanomaterial factors that affect the EPR effect to achieve the therapeutic effect. In general, tumors possess the following four main pathophysiological characteristics: 1) extensive angiogenesis, 2) lymphatic drainage/recovery system, 3) significantly increased production of permeability mediators, 4) abnormal vasculature that has poorly aligned endothelial cells, impaired functional receptors of angiotensin II and impaired lymphatic system, and lacks smooth muscle layer. Researchers take advantage of these unique tumor features to improve the efficacy of the targeting drug delivery systems (TDDSs). Besides, size, shape, surface charge, and surface wettability play an essential role in drug delivery (Dai et al., 2016).

So far, nanocarriers are classified into five types: lipid-based nanoparticles (liposomes), polymer-based nanoparticles and micelles, dendrimers, carbon-based nanoparticles, and metallic and magnetic nanoparticles. Liposomes are self-assembled, uni-lamellar, or multi-lamellar spherical vesicles ranging in size from 20 to 1000 nm (Torchilin, 2005; Samad et al., 2007), which have the following advantages: 1) encapsulate both hydrophilic and hydrophobic therapeutic agents with high efficiency; 2) protect the encapsulated drugs from the unfavorable effects of external conditions; and 3) be functionalized to obtain various benefits (e.g., ligand-mediated specific targeting, formation of the desired composition, and prolongation of circulation half-life) (Zhang et al., 2008). Polymeric micelles are self-assembled, monolayer systems formed spontaneously under suitable conditions, which can prevent the uptake of the mononuclear phagocyte system as well as evade renal excretion due to their hydrophilic corona and small size (<100 nm), respectively (Sahoo and Labhasetwar, 2003; Kumari et al., 2016). Dendrimers are polymeric molecules with defined molecular weights, large numbers of functional groups on the surface, and well-established host-guest entrapment properties. They are composed of multiple perfectly branched monomers that emanate radially from a central core (Lee et al., 2005). Carbon-based nanocarriers, such as carbon nanotubes (CNTs), can be considered tubes rolled from layers of graphene sheets. One of its notable advantages is that CNTs can easily penetrate all kinds of cells, even hard-to-transfect cells (Kumari et al., 2016). Metallic nanoparticles can be synthesized and modified with various chemical functional groups that allow them to be conjugated with antibodies, ligands, and drugs of interest. Their value has been manifested in biomedical applications such as magnetic separation, targeted drug delivery, and diagnostic imaging. But on the other hand, their biocompatibility and toxicity limit their utilization *in vivo* (Mody et al., 2010). Magnetic particles have a promising potential in targeting drug delivery, serving as imaging contrast agents, and contaminated water treatment (Corot et al., 2006; Punia et al., 2021).

Although PTDDSs have superiority over conventional chemotherapeutic drugs, in terms of increased circulation time *in vivo*, reduced uptake of endotheliocytes and phagocytes, improved drug efficacy, and so on, they still have many defects and face multiple challenges requiring further study to overcome.

### 2.2 Active targeted drug delivery systems

Active targeted drug delivery systems (ATDDs) are based on the interaction of attached high-affinity ligands and cognate receptors, and the ligands can selectively bind to receptors on the target cells. A variety of ligands have been used in ATDDSs, such as the above-mentioned small molecules (e.g., FA), macromolecules (e.g., peptides), and so on (Wicki et al., 2015). Hence, they can also be regarded as ligand-mediated targeting nanocarriers. ATDDSs can reduce off-target effects and improve the bioavailability of chemotherapeutic agents. More importantly, some advanced active drug carriers can achieve environment-responsive drug delivery or controllable drug delivery and transport nanocarriers to the target sites when exposed to endogenous (pH, hypoxia, etc.) and exogenous stimuli (e.g., ultrasound, light, heat, and magnetic field) (Dai et al., 2016). The advantages mentioned above show that ATDDSs have been extensively investigated and drawn much attention.

TDDSs based on nanomaterials are an emerging method for cancer treatment. The properties of nanocarriers, including their nanoscale sizes, high surface-to-volume ratios, favorable drug release profiles, and targeted modifications, can enable them to reach target tumor tissues better and release drugs in a stable, controlled manner. However, the traditional targeted drug delivery systems still confront some problems and challenges. Firstly, nanocarriers may interact with biological fluid (e.g., blood serum) or components (e.g., proteins) that exist in the bio-environment, and their structures, sizes, surface properties, and charges are unfavorably changed, significantly influencing the function of nanomedicine. The second challenge is to precisely evaluate nanocarriers’ toxicity because of many variables that hinder the characterization, such as material, size, shape, surface area, surface charge, porosity, and hydrophobicity. The third difficulty is detecting their distribution and location in real-time (Wicki et al., 2015). Additionally, biostability, clearance rate of nanocarriers, and tolerance of the body also stand in the way of their clinical translation (Dai et al., 2016). More importantly, the developed PTDDSs and ATDDSs have no self-propulsion capability and only show a low tumor targeting efficiency (0.7%, median) because of their passive diffusion and short-range recognition (<0.5 nm) targeting strategies.

## 3 Micro/nanorobots

Different from ordinary nanomaterials, MNRs can realize self-propulsion by converting the surrounding energy into mechanical motion, thus breaking the constraints of irregular Brownian motion and low Reynolds number on the movement of micro/nanoscale objects. The propulsive force helps MNRs to traverse biological barriers such as the dense extracellular matrix, blood-brain barrier, and blood-tumor barrier in an autonomous manner (Joseph et al., 2017; Wang et al., 2019; Zhang et al., 2021), rather than passively circulating through blood flow. The tumor-targeted delivery capability of MNRs can effectively improve drug bioavailability and reduce the required drug dose to minimize side effects (Alapan et al., 2020; Zhou et al., 2021). In the following part, we classify and demonstrate the MNRs with different driving mechanisms according to the power source.

### 3.1 Chemically-powered micro/nanorobots

**FIGURE 2:**
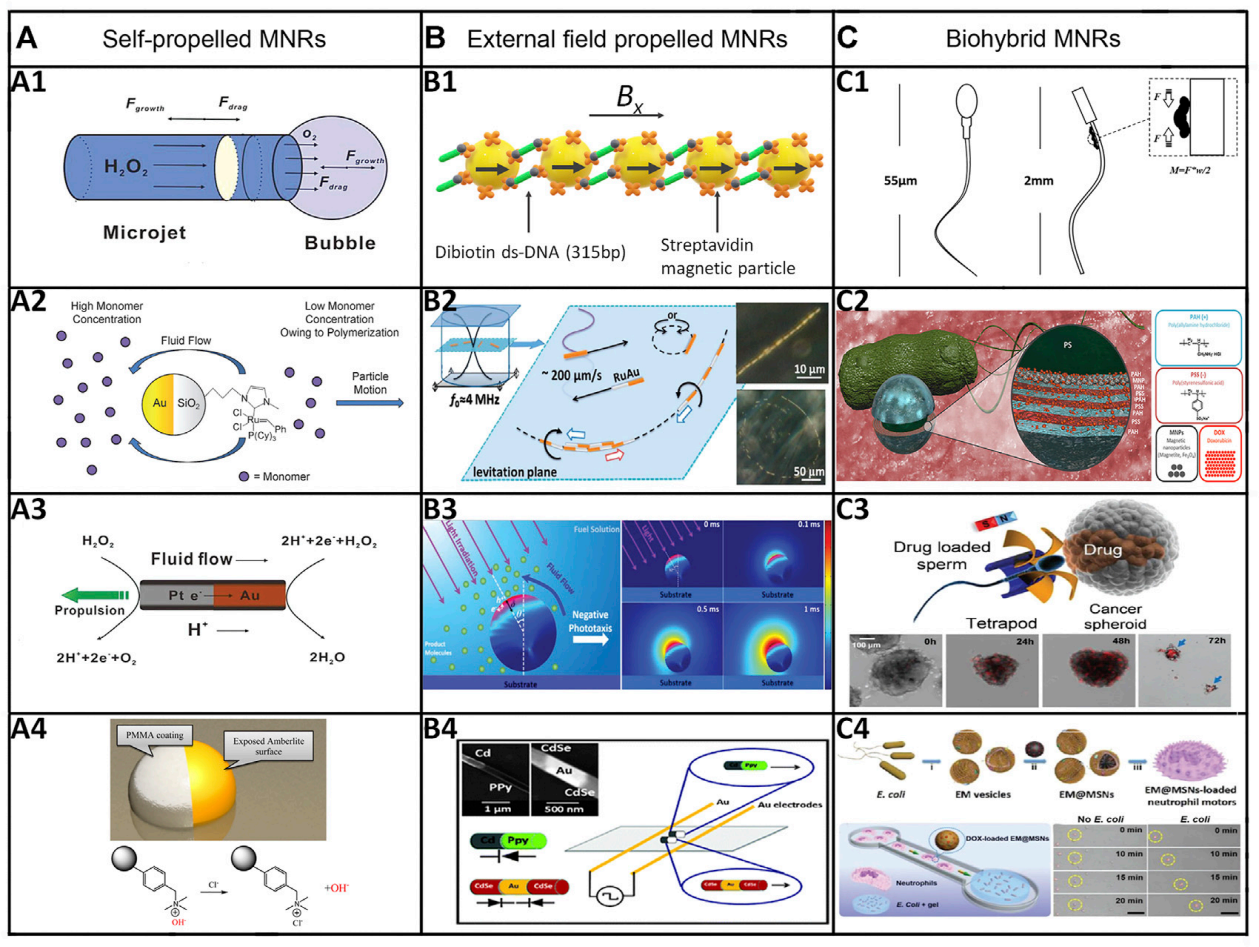
Categories and representative examples of MNRs. **(A)** Chemically-powered MNRs. (A1) Bubble propulsion: tubular catalytic microrobot propelled by O_2_-bubble ejection in an aqueous H_2_O_2_ solution. (Manjare et al., 2013) Copyright 2013. Reproduced with permission from American Chemical Society. (A2) Self-diffusiophoresis: polymerization-powered microrobot. (Pavlick et al., 2011) Copyright 2011. Reproduced with permission from John Wiley and Sons. (A3) Self-electrophoresis: Au‒Pt bimetallic nanorod propelled by a self-electrophoresis mechanism in an aqueous H_2_O_2_ solution (Paxton et al., 2004). Copyright 2004. Reproduced with permission from American Chemical Society. (A4) Surface tension gradient: autonomous motors powered by the rapid depolymerization reaction of poly (2-ethyl cyanoacrylate) (PECA). (Zhang et al., 2013) Copyright 2013. Reproduced with permission from American Chemical Society. **(B)** External-field-powered MNRs. (B1) Magnetic field: magnetic field propelled micromotor (Dreyfus et al., 2005). (B2) Ultrasound: metallic micro-rods propelled by ultrasound. (Wang et al., 2012) Copyright 2012. Reproduced with permission from American Chemical Society. (B3) Light: an isotropic TiO_2_ microrobot powered by UV light. (Chen et al., 2017) Copyright 2017. Reproduced with permission from John Wiley and Sons. (B4) Electric field: rod-like nanorobots powered by an electric field. (Calvo-Marzal et al., 2010) Copyright 2010. Reproduced with permission from The Royal Society of Chemistry. **(C)** Biohybrid MNRs. (C1) Muscle cells: a muscle-cell-driven hybrid microrobot (Williams et al., 2014) (C2) Bacteria: bacteria-driven microswimmers based on polyelectrolyte multilayer (PEM) magnetic nanoparticles attached to an *E. coli MG1655* bacterium. (Park et al., 2017) Copyright 2017. Reproduced with permission from American Chemical Society. (C3) Sperms: sperm-hybrid microrobots for drug delivery in the female reproductive tract. (Xu et al., 2018) Copyright 2018. Reproduced with permission from American Chemical Society. (C4) Immune cells: nanoparticle-loaded neutrophil micromotors. (Shao et al., 2017) Copyright 2017. Reproduced with permission from John Wiley and Sons.

Chemically-powered MNRs are those MNRs that can move on their own by converting chemical energy into mechanical driving forces (Luo et al., 2018). After decades of development, there have been multiple choices of usable materials, geometries, and surface modifications that can be applied to their designs, which provide us with an abundant variety of existing MNRs. The following paragraph will give a summary demonstration of four major approaches used to achieve chemically-powered self-propulsion (Figure 2A).

The bubble-propelled MNRs are propelled by the recoil effect of the ejection of bubbles generated from the reaction between the body material and the bio chemical fuel, which generally consists of peroxide and bioactive fluids (Manjare et al., 2013). Considering the notable high-speed feature of bubble-propelled MNRs, researchers have put a lot of effort into applying them in the biomedical field. Diffusiophoresis has two types: electrolyte (ionic) and non-electrolyte (non-ionic) diffusiophoresis. The self-diffusiophoretic MNRs can propel themselves by a self-generated concentration gradient, which is mainly produced by chemical reactions on the surface that consume reactants and generate products (Pavlick et al., 2011; Zhang et al., 2013). Unlike ordinary electrophoresis, self-electrophoresis-driven MNRs usually do not directly respond to external electric fields. Instead, they generate a local electric field through chemical gradients and move in response to the self-generated electric field caused by the asymmetric distribution of ions (Paxton et al., 2004). The surface tension gradient can be produced by accomplishing an asymmetric release of a preformulated chemical from the MNRs and composing the formulation of the solution media. The interfacial energy gradient can easily cause a mass flow, which can efficiently propel those micro/nano-sized objects, referred to as the Marangoni effect (Zhang et al., 2013; Yang et al., 2020).

Many chemically-powered MNRs are based on the catalytic reaction of H_2_O_2_. The biological toxicity of H_2_O_2_ significantly limits the biomedical application of these MNRs. Hence, researchers began to look for alternative fuels to power MNRs. Among them, glucose, urea, water, and other existing natural substances in the body have been widely studied. For example, Mou et al. proposed employing Mg-water and Mg-body fluid reactions to construct biocompatible bubble-propelled Mg-based micromotors (Mou et al., 2013; Mou et al., 2014; Xiong et al., 2020). Then, de Avila et al. further demonstrated that Mg-based MNRs could operate *in vivo* (Esteban-Fernandez de Avila et al., 2017). The surface of the motor was coated with a poly (lactic-co-glycolic acid) (PLGA) layer and a chitosan layer containing clarithromycin (CLR), which could exercise and treat gastritis with gastric acid as fuel in the mouse model. Mg in the micromotor can react with H^+^ in gastric juice to generate hydrogen bubbles to propel the motor and increase the pH value of gastric juice. The dissolution of the Mg core in the motor leads to motor degradation and drug release. At the same time, the chitosan coating with a positive charge can make the motor adhere to the gastric wall and promote the effective local release of drugs in PLGA. In addition, enzymatic MNRs can employ bioavailable chemicals (e.g., glucose and urea) as biocompatible fuels. As reported by Sanchez et al., urease-loaded silica microbots can catalyze the decomposition of urea into ammonia and carbon dioxide so that they can move autonomously (Hortelao et al., 2018; Liu et al., 2022). Furthermore, experimental results have proved that they can function normally even in ionic media (phosphate buffer solution, PBS), and are capable of loading a large amount of anti-cancer drugs (Doxorubicin, DOX) and actively transporting them toward cancer cells, which is very important for their application in cancer treatment. Very recently, Mou and co-workers found that ZnO-based micromotors can not only be powered by an ultra-low level of CO_2_, but also show highly efficient chemotaxis toward a CO_2_ source because of their high sensitivity to CO_2_ dissolved in water (Mou et al., 2021). Thus, they may be able to “seek out” specific cells or pathogen microorganisms and execute targeted biomedical operations by autonomously tracking the metabolic CO_2_ signals emitted from them.

### 3.2 External-field-powered micro/nanorobots

External-field-powered MNRs refer to fuel-free MNRs that are actuated by light, ultrasound, electric field, or magnetic field. Compared with chemically-powered MNRs, external-field-powered MNRs have the advantages of better controllability, a long lifetime, and less harmful undesired impact, so they have a broader application prospect (Figure 2B).

Magnetic field-driven MNRs are mainly powered by alternating magnetic fields (i.e., rotating, oscillating, and on-off magnetic fields) (Peyer et al., 2012; Wang B et al., 2020; de la Asuncion-Nadal et al., 2022). Generally, magnetic field-driven MNRs are moving in two ways. First, MNRs can move in low Reynolds number fluids by deforming their bodies in a non-reciprocal way when applied to a rotating or oscillating magnetic field. For example, helical microrobots can turn clockwise, corresponding to the clockwise rotating magnetic field, and transform the rotation around their helical axis into a translational corkscrew motion (Yu et al., 2017). Second, magnetic fields can propel MNRs by producing an asymmetric force field in the vicinity, as MNRs experience more significant hydrodynamic drag near the wall surface than the side further away from it (Chen et al., 2019).

Ultrasound has drawn much attention as a biocompatible, powerful energy source easily accessible in hospitals and laboratories. Generally, the interdigital transducer and bulk acoustic wave devices can generate surface and ultrasonic standing waves, respectively. When exposed to an ultrasound field, the MNRs would experience acoustic radiation forces, which consist of primary radiation force (PRF), the leading force in the field of acoustic waves that is responsible for the migration of MNRs, and secondary radiation force (SRF), the weaker force than the axial PRF that is responsible for the repulsion and attraction between MNRs (Xu et al., 2017). Researchers have proposed three mechanisms of ultrasound-based propulsion. The first one is the self-acoustophoresis mechanism that applies to metallic nanowires, which claims that the asymmetry of composition or shape of the nanorods can cause random directional movement when under the ultrasound field. The second mechanism can be demonstrated *via* an example—Wang et al. developed a hollow conically shaped microcannon loaded with nanobullets made of silica or fluorescent nanospheres. Under ultrasound, the perfluorocarbon emulsion on the inner surface of the microcannon goes through instantaneous evaporation. It results in the rapid ejection of nanobullets, which allows the microcannon to travel at high speed (Wang et al., 2012). Such a feature has the potential to be used in tissue penetrating. The last one applies to MNRs trapped with air bubbles. The applied ultrasound field can lead to the formation of acoustic streams and propel them (Soto et al., 2016). In addition to using the ultrasound field to propel MNRs, it has also been used to control the motion of MNRs (such as bubble-propelled microtubes) and induce swarming behaviors (Fernandez-Medina et al., 2020).

As a green energy source, light demonstrates its merits in actuating MNRs, controlling motion, conducting collective behavior, etc. The autonomous motion of MNRs can be achieved by three main approaches, which are light-induced physical effects (e.g., photothermal effect, Marangoni effect, and momentum transfer), light-induced chemical reactions, and light-induced body deformation (Mourran et al., 2017; Safdar et al., 2017; Xu et al., 2017). Hence, to generate motion, light-driven MNRs are required to either produce a local non-uniform gradient field or periodically perform body deformation (Wang et al., 2012; Chen et al., 2017). The non-uniform gradient field can be generated by either applying the non-uniform light field or designing the asymmetric structure (Wang et al., 2020). For example, single or swarming isotropic TiO_2_ micromotors can be propelled under UV irradiation utilizing the asymmetric photocatalytic reactions and chemical gradients across their illuminated and shadowed sides (Mou et al., 2019; Liang et al., 2020; Che et al., 2022). As for motion caused by body deformation, the motion is inspired by microorganisms in nature [e.g., metachrony of ciliates, whole body deformation of marine phytoplankton, rotation of *Escherichia coli* (*E. coli*), etc.] and comes from the interaction of the photo-responsive material with light, with little or no dependence on the properties of surroundings (e.g., ionic strength of the surrounding medium). There are a variety of motion modes for this type of biomimetic morphing microswimmers based on different geometric designs and applied light fields. For instance, Palagi et al. have reported that a simple-shaped cylindrical microrobot can generate traveling-wave body deformation and propel opposite the wave direction under structured monochromatic light with a periodic traveling pattern (Palagi et al., 2016).

Electric fields can also be an energy source for MNRs, and four standard methods exist to utilize them. The first way is to apply the alternating current (AC) electric field to polarize the metal-dielectric Janus microparticle differently, which further leads to asymmetric electroosmotic flows that propel the microparticle moving away from the metal side. The second way takes advantage of the diode’s ability to rectify the AC electric field into a direct current electric field, which further induces electrokinetic flows and achieves self-actuation. The third way is to use the electric field to cause chemical reactions. The last method takes advantage of the Quincke effect. An AC electric field can induce the electrokinetic effect that allows dielectric micromotors to rotate and move to the targeting site in the liquid medium of low conductivity (Chen et al., 2019). Although electrically driven MNRs are more easily manipulated by electric tweezers with high precision and versatility, the bioapplications of electric field-propelled MNRs may be limited due to their short locomotion range and the lack of biocompatibility in high ionic bio-medium (Calvo-Marzal et al., 2010; Wang and Gao, 2012; Wang et al., 2021).

### 3.3 Biohybrid micro/nanorobots

Biohybrid MNRs can be developed by merging non-living systems with biological components at various scales of molecules, cells, organisms, and tissues, to obtain desirable functions that integrate the advantageous features of living biological materials (e.g., high energy efficiency, high power-to-weight ratio, ample energy storage, environmental biocompatibility, self-repair, and self-assembly) and non-living systems (e.g., high accuracy, high strength, favorable repeatability, and controllability) (Williams et al., 2014; Park et al., 2017; Shao et al., 2017; Xu et al., 2018; Zhang et al., 2018; Gao et al., 2021) (Figure 2C). To develop biohybrid MNRs that can display biomimetic behavior and execute on-demand tasks, proper structure design, functional modification (e.g., ligands, antibodies), and employment of powerful actuators (e.g., living cells, organisms) are requisite. For instance, Xu and coworkers developed a cellular engine, “muscular fin,” utilizing NIR light irradiation (Xu B et al., 2019). Another interesting study was carried out by Sun and coworkers. They presented the cardiomyocyte-driven soft robots composed of claws similar to snakeskin, a parallel CNT-assisted myocardial tissue layer, and a structural color indicator layer. The orientation-inducing and electrical properties of the parallel-aligned-CNT layer facilitate the regular beating ability and contraction performance of cardiomyocytes; the asymmetric claws can assist soft robots in accomplishing directional movement by acting as supporting points to provide the necessary friction; the structural color-indicator layer can be used for monitoring the motion state. Regarding the advantages mentioned above, the biohybrid MNRs can be integrated with a microfluidic system to evaluate drug screening (Wan et al., 2019). Other notable research about biohybrid MNRs has also been conducted recently (Aydin et al., 2019; Hasebe et al., 2019; Li et al., 2019; Yamatsuta et al., 2019).

Despite the tremendous progress of synthetic MNRs, many challenges still hinder the medical application of these MNRs. Biological hybrid drug delivery platforms stand out with the potential to become the ideal drug delivery candidate in future medical applications. Because of their high physiological adaptability and functional immunosuppression, engineered biological motile organisms or cells have demonstrated exceptional abilities to protect loaded drugs or prevent them from causing severe side effects on normal cells or tissues by encapsulating drugs inside them. Moreover, their high biocompatibility and cell affinity facilitate increasing the drug uptake rate of lesions. In addition, they can respond to both exogenous (i.e., nutrients, oxygen, and pH) and endogenous (light, ultrasound, magnetic fields, and electric fields) stimuli to achieve targeted delivery or drug release.

Organisms in nature have evolved into multiple efficient actuating systems (Sun et al., 2020) that can flexibly self-propel and adapt to the changing environment within a specific range. For instance, bacteria can ingest nutrients in the environment and convert them into energy to propel themselves. Within a certain range, bacteria can conduct the corresponding adjustments according to environmental change to better adapt to the environment and facilitate their survival. The combination of chemotaxis and external field guidance can accurately lead them to the targeting sites and release drugs. Researchers have combined one or more bacterial strains with magnetic microparticles or nanoparticles. When exposed to an external magnetic field, these magnetic particles will be displaced in the direction of the magnetic field. Subsequently, the bacteria will also move accordingly in the direction of the magnetic field. In 2017, Sitti and colleagues attached *E. coli* to microparticles composed of the DOX and a layer of tiny magnetic nanoparticles. The researchers placed cancer cells in a Petri dish and discovered through research that they could remotely control the movement of these drug-carrying bacterial robots with magnets. This solution can improve the targeting of drugs to tumor cells compared with the method of only adding drug microparticles to tumor cells (Park et al., 2017).

As for sperm-based biohybrid MNRs, their excellent biological characteristics are crucial for medical application. Sperms can provide MNRs with strong driving forces to move through viscous biofluids as well as enough space to load therapeutic drugs or imaging agents. Secondly, apart from several substances on the membrane that can protect loaded drugs from clearance by immunosuppression. Their unique acrosome structure was confirmed to induce cell fusion between sperm and somatic cells, which is beneficial for the drug uptake of target cells. For example, as shown in Figure 2C, the drug-loaded sperm is coupled to the magnetic tetrapod-like microtube. It can be steered to the targeting sites under the guidance of the magnetic field. When this microrobot hits the tumor spheroid, the sperm squeezes through the cancer cell and fuses with the cell membrane, resulting in drug release into the target cell (Xu et al., 2018). These sperm-biohybrid MNRs media are promising to be applied for drug delivery of cancer treatment or other diseases in the reproductive system.

In recent years, biohybrid MNRs have received much attention and exhibited remarkable experimental results *in vitro* and in animal models. However, there remain many challenges to be overcome. The way to maintain viability and stability in a complex bio-environment and the approach to prolonging their lives are essential for their medical application.

## 4 Motile drug delivery by micro/nanorobots

Existing TDDSs mainly include passive targeting based on the EPR effect and active targeting based on molecular recognition (identification distance <0.5 nm). One of the biggest problems is drug delivery efficiency: 0.7% (median) of the administered nanoparticle dose is found to be delivered to a solid tumor (Wilhelm et al., 2016). Rapid blood flow, the mononuclear phagocyte system, and renal clearance are obstacles to tumor therapy. Moreover, the complex tumor environment hinders many targeted drug delivery systems, such as high interstitial fluid pressure that hampers tumor tissue penetration; hypoxia conditions that may limit some therapeutic agents; dense extracellular matrix; and abnormal tumor vasculature (Li et al., 2020). The challenges mentioned above make it very difficult to achieve effective treatment outcomes. Developing “motile-targeting” drug delivery platforms based on MNRs is essential to improve anti-tumor efficiency and reduce toxic and side effects, and is also a hot spot in tumor therapy research. Generally, the motile microcarriers based on microrobots exhibit a strong driving force, high directionality, and high drug-loading capacity, and thus they are expected to deliver and manipulate heavy cargoes (e.g., cells and drug capsules) in complex bio-environments. As the size of MNRs decreases to the nanoscale range, they may suffer from random superdiffusive motions in bio-media because the stochastic Brownian effect gradually dominates the motion dynamics, but show unique advantages in overcoming cellular barriers and internalization into cells when executing drug delivery. Therefore, both the motile micro- and nanocarriers based on MNRs are promising candidates to perform “motile-targeting” drug delivery.

To load drugs in MNRs, three key strategies are usually adopted, including post-loading, co-loading, and pre-loading. An appropriate drug loading method could be selected according to the specific properties of a particular nanocarrier (material, structure, surface properties, etc.). The post-loading strategy usually refers to the drug loading of the pre-synthesized porous nanocarriers by mixing with drug solutions, enabling the drugs to be loaded onto the nanocarriers through various mechanisms such as adsorption, electrostatic interactions, entrapment, and hydrophobic forces. The co-loading method usually involves coupling drugs with polymers or macromolecules, and then the drug conjugates self-assemble to form drug-loaded nanocarriers. The pre-loading strategy first forms drug nanoparticles and coats them with a layer of other materials to form nanocarriers with drug core and protective shell structures. After loading drugs, MNRs can move towards the targeting sites and then realize targeted drug release under the guidance of endogenous (i.e., chemotaxis) or exogenous stimuli (i.e., electric field, magnetic field, ultrasound, or light) (Medina-Sanchez et al., 2018; Soto et al., 2020) (Figure 1).

### 4.1 Chemically-powered micro/nanorobots

Due to the autonomous motions and possible intelligent chemotaxis, chemically-powered MNRs have been widely explored for active drug delivery in tumor therapy and have made significant progress in the past few years. The representative examples are summarized in Table 1.

**TABLE 1:** Chemically-powered MNRs for motile-targeting drug delivery.

Energy sources	Composition	Environment	Motion behavior	Loaded cargo/therapeutic drugs	Biomedical application	Ref.
H_2_O_2_	CNTDOX-Fe_3_O_4_-Tf/COX-Fe_3_O_4_-mAb nanobots	*In vitro*	PBS: 0.338 mm/s, DMEM: 0.831 mm/s, blood serum: 1.011 mm/s (0.5% H_2_O_2_)	DOX hydrochloride	Chemotherapy for tumor	Andhari et al. (2020)
	Dual-drive hybrid micromotors (PS@Fe_3_O_4_@Pt-PS)	*In vitro*	≈12.5 μm/s (10% H_2_O_2_)	N.A.	Drug delivery in future	Chen et al. (2020)
	Graphene/FeOx-MnO_2_ micromotors	*In vitro*	Average speed 89 ± 59 μm/s (0.03% H_2_O_2_)	N.A.	N.A.	Ye et al. (2018)
	PEG-PS polymersome-based Janus nanomotors	*In vitro*	N.A.	Fluorescein sodium salt (model drug)	Drug delivery	Peng et al. (2018)
Water	PACT-guided microrobotic system	*In vitro*, *In vivo*	< 1 mm/min	DOX	Drug delivery	Wu Z et al. (2019)
	Qβ VLPs-loaded Mg-based micromotors	*In vitro*, *In vivo*	Average speed in intraperitoneal (IP) fluid ≈60 μm/s	Qβ VLPs	Cancer immunotherapy (ovarian cancer)	Wang Cet al., (2020)
	Mg-Fe_3_O_4_-based Magneto-fluorescent nanorobots	*In vitro*	0.393 ± 0.07 mm/s in serum with 1.0 M NaHCO_3_	N.A.	Capture and isolate tumor cells	Wavhale et al. (2021)
L-arginine	NO-driven nanomotors	*In vitro*	HLA10: 3 μm/s, HLA15:8 μm/s, HLA20:13 μm/s	NO, HPAM, L-citrulline	Various diseases (e.g., tumor)	Wan et al. (2019)
Native acid	Calcium carbonate micromotors	*In vitro*	0.544 μm/s	N.A.	Drug delivery for cancer treatment	Guix et al. (2016)
	Micromotor toxoids	*In vitro*, *In vivo*	∼200 μm/s	Antigen	Gastrointestinal drug delivery	Karshalev et al. (2019)
	Macrophage-Magnesium biohybrid micromotors	*In vitro*	Average speed ≈127.3 μm/s	N.A.	Endotoxin neutralization	Zhang et al. (2019)
	Poly (aspartic acid)/iron−zinc microrockets	*In vitro, In vivo*	≈29.2 ± 7.9 μm/s (gastric acid simulant)	DOX	Chemotherapy (gastric cancer)	Zhou et al. (2019)
Collagen (collagenase)	Collagenase-powered MF-NPs coated microswimmers	*In vitro*	≈22 μm/s (collagen solution)	Multifunctional nanoparticles	Potential for Cargo delivery	Ramos-Docampo et al. (2019)
H_2_O_2_ (catalase)	Ultrasmall stomatocyte polymersomes	*In vitro*	From 13.69 ± 1.11 to 20.52 ± 0.35 μm/s (2–20 mM H_2_O_2_)	N.A.	Potential for cargo delivery	Sun et al. (2019)
Glucose (GOx)	Dual enzyme-functionalized core-shell nanomotors	*In vitro*	N.A.	Photosensitizer, upconversion nanoparticles	Synergetic photodynamic and starvation therapy	You et al. (2019)
Urea (urease)	enzyme-powered Janus platelet micromotors	*In vitro*	≈7 μm/s (200 mM urea concentration)	DOX	Various disease (e.g., breast cancer)	Tang et al. (2020)
	Multilayer-urea -based Janus Au/MMPs	*In vitro*	21.5 ± 0.8 μm/s (physiological urea concentrations (10 mM))	N.A.	Potential for drug delivery	Luo et al. (2020)
	Urease-powered silica NPs based nanomotors	*In vitro*	N.A.	N.A.	Targeted bladder cancer therapy	Hortelao et al. (2018)
	Enzyme-powered gated mesoporous silica nanomotors	*In vitro*	N.A.	DOX, [Ru (bpy)_3_]Cl_2_ (bpy = 2,2′-bipyridine)	Intracellular Payload Delivery	Llopis-Lorente et al. (2019b)

#### 4.1.1 Peroxide-powered micro/nanorobots

**FIGURE 3:**
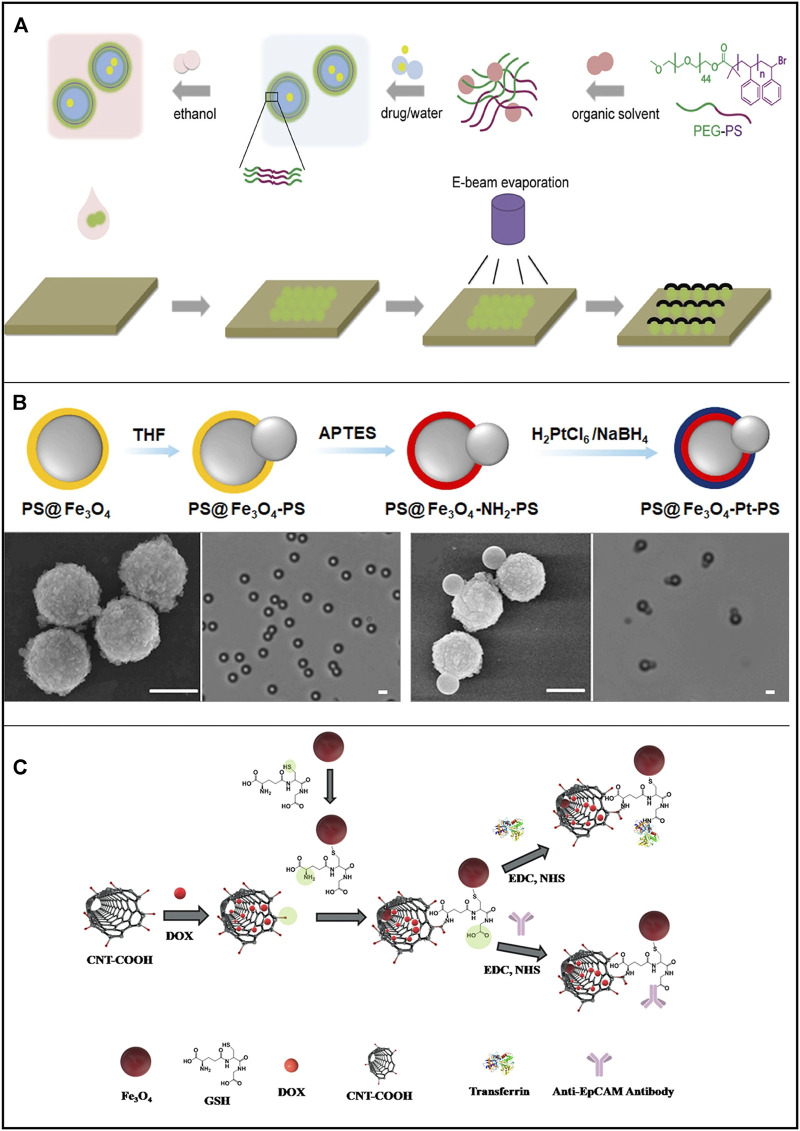
Motile drug delivery by peroxide-powered MNRs. **(A)** Poly (ethylene glycol)-b-polystyrene (PEG-PS) polymersome-based Janus nanomotors. (Peng et al., 2018) Copyright 2018. Reproduced with permission from John Wiley and Sons. **(B)** Dual-drive hybrid micromotors (PS@Fe_3_O_4_@Pt-PS). (Chen et al., 2020) Copyright 2020. Reproduced with permission from Elsevier Inc. **(C)** Multi-component magnetic nanobot designed by chemically conjugating magnetic Fe_3_O_4_ nanoparticles (NPs), anti-epithelial cell adhesion molecule antibody (anti-EpCAM mAb) to multiwalled carbon nanotubes (CNT) loaded with DOX. (Andhari et al., 2020) Copyright 2020. The Authors, some rights reserved; exclusive licensee Springer Nature.

Peroxide-based MNRs have been extensively studied and have made significant progress in revealing the propelling mechanisms, motion behaviors, and cargo delivery processes since the beginning of MNR research. Peng and coworkers proposed a polymersome nanomotor-based strategy to enhance ERP in drug delivery (Peng et al., 2018). The straightforward fabrication process is as follows: The amphiphilic di-block copolymer poly (ethylene glycol)-b-polystyrene (PEG-b-PS) self-assembles into tunable-size vesicles, which are then loaded with the hydrophilic model cargo fluorescein sodium salt, and the catalytic platinum cap is partially deposited on the surface of the polymersome *via* electron beam evaporation (Figure 3A). The nanomotor is based on the bubble-propelled mechanism and can be triggered under ultrasound stimuli to release the encapsulated model cargo. In a tumor vasculature model, these cargo-loaded polymersome nanomotors demonstrated enhanced penetration across the endothelium. Besides, Chen and coworkers developed a simple method to prepare dual-drive hybrid micromotors for drug delivery (Chen et al., 2020) (Figure 3B). They use magnetic polystyrene particles (MPS) to synthesize snowman-shaped particles (PS@Fe_3_O_4_-PS) by swelling them in a suitable solvent and then introducing platinum on the surface of the Fe_3_O_4_ components by selective surface modification. Thus, such hybrid MNRs can be powered by both chemical reactions (platinum-catalyzed decomposition of H_2_O_2_) in the presence of H_2_O_2_ and an external magnetic field (a magnetic gradient field exerts force through Fe_3_O_4_). Andhari et al. fabricated multi-component magnetic nanorobots by chemically conjugating magnetic Fe_3_O_4_ nanoparticles with anti-epithelial cell adhesion molecule antibody (anti-EpCAM mAb) to multiwalled CNTs loaded with an anticancer drug (i.e., DOX) (Figure 3C) (Andhari et al., 2020). The nanorobots are propelled by the O_2_ bubbles generated by the Fe_3_O_4_ nanoparticle-catalyzed decomposition of H_2_O_2_ and can be steered by the external magnetic field. The average propulsion speed of the CNT-DOX-Fe_3_O_4_-Tf nanobot during its upward movement velocity in PBS, DMEM, and the blood serum was 0.338, 0.831, and 1.011 mm/s, respectively, in 0.5% H_2_O_2_. In addition, the nanobots preferably release DOX in the intracellular lysosomal compartment of human colorectal carcinoma (HCT116) cells by opening the Fe_3_O_4_ nanoparticle gate. Further, nanobots reduce *ex vivo* HCT116 tumor spheroids more efficiently than free DOX.

Peroxide-based MNRs can generate sufficient driving force to propel themselves in different media (i.e., diluted hydrogen peroxide solution, PBS, blood serum, etc.) and realize controlled cargo delivery. Nonetheless, hydrogen peroxide is a toxic fuel for the human body, and further effort is needed to explore its biocompatible counterparts.

#### 4.1.2 Biocompatible fuel-powered micro/nanorobots

Most of the reported chemically-driven MNRs are unsuitable for biomedical use as they rely on fuels that are incompatible with living organisms (e.g., H_2_O_2_) for chemical propulsion. Hence, there is a great need to develop MNRs driven by biocompatible fuels that can be better used for different biomedical applications. Biofluids, including blood, urine, gastric juice, pancreatic juice, breast milk, etc., may be reactive with active metals (e.g., Mg, Zn, and Fe), oxides (e.g., ZnO), or carbonates (e.g., CaCO_3_). Motivated by this, biofluid-powered MNRs have been developed recently.

**FIGURE 4:**
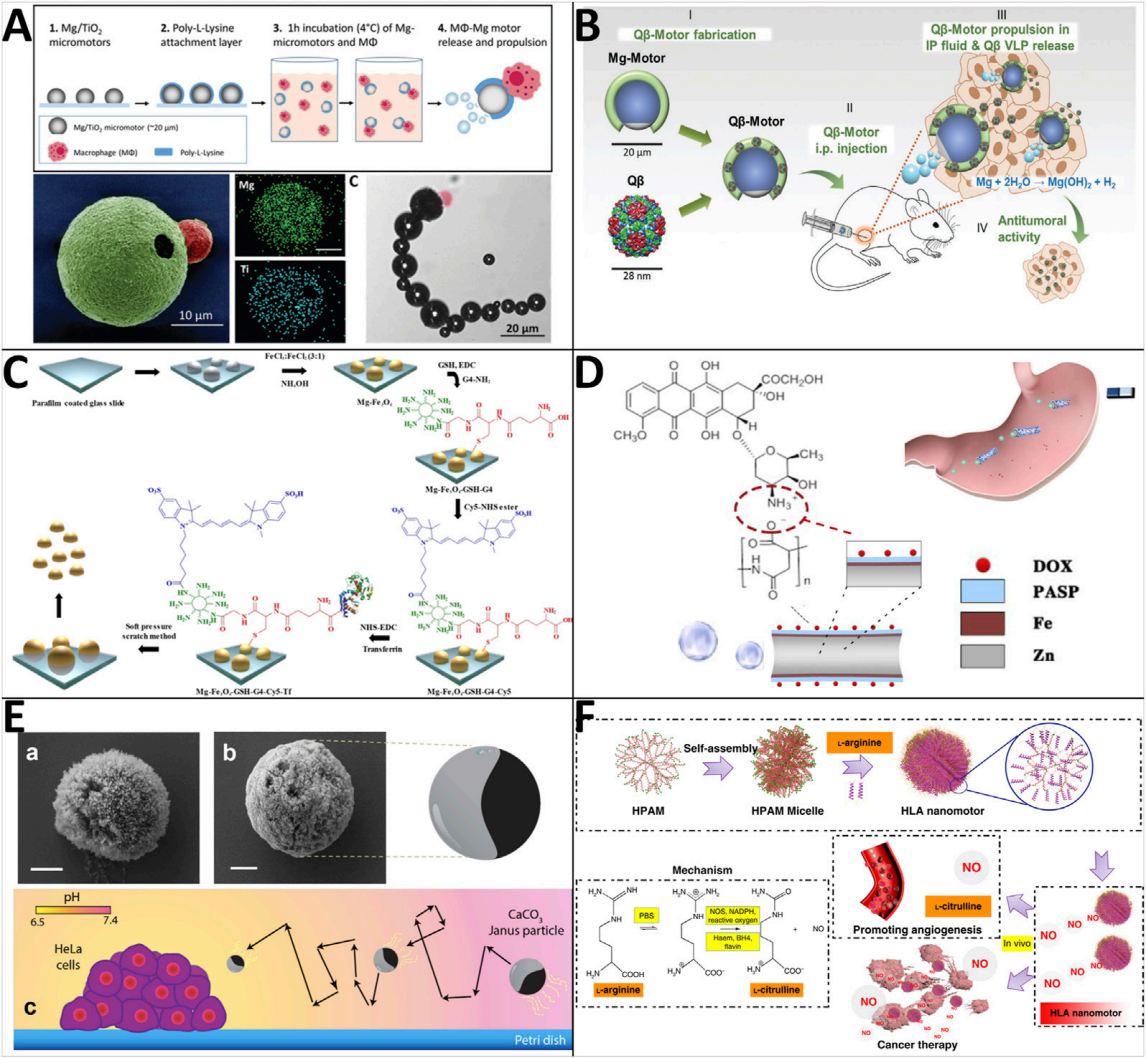
Motile drug delivery by biocompatible fuel-powered MNRs. **(A)** Macrophage-magnesium biohybrid micromotor. (Zhang et al., 2019) Copyright 2019. Reproduced with permission from John Wiley and Sons. **(B)** Bacteriophage virus-like nanoparticles (QβVLPs)-loaded Mg-based micromotors. (Wang C et al., 2020) Copyright 2020. Reproduced with permission from John Wiley and Sons. **(C)** Mg-Fe_3_O_4_-based Magneto-fluorescent nanorobot. (Wavhale et al., 2021) Copyright 2021. The Authors, some rights reserved; exclusive licensee Springer Nature. **(D)** Poly (aspartic acid)/iron-zinc microrockets. (Zhou et al., 2019) Copyright 2019. Reproduced with permission from American Chemical Society. **(E)** Calcium carbonate micromotors. (Guix et al., 2016) Copyright 2016. The Authors, some rights reserved; exclusive licensee Springer Nature. **(F)** NO-driven nanomotors. (Wan et al., 2019) Copyright 2019. Reproduced with permission from Springer Nature.

Early in 2013, Mou et al. found that Mg-based micromotors could directly use water and blood plasma as fuels to realize efficient bubble-propelled self-propulsion (Mou et al., 2013; Mou et al., 2014). Inspired by this phenomenon, various biomedical Mg-based microrobots have been developed. For instance, Zhang et al. reported that live cells could be integrated with synthetic components to endow MNRs with remarkable capabilities for biomedical applications (Zhang et al., 2019). The macrophage-magnesium micromotors are fabricated by coating Mg microparticles with titanium dioxide (TiO_2_) and a poly (l-lysine) (PLL) layer and attaching the macrophage to the outer PLL coating *via* electrostatic interactions (Figure 4A). Cell membrane staining and toxin neutralization studies confirm that the macrophages maintain their viability and functionality (e.g., endotoxin neutralization) after binding to the Mg micromotors. Additionally, they can move in simulated gastric acid at an average speed of 127.3 μm/s and neutralize nearly 66% of endotoxin in 10 min. Immunotherapy is emerging as an attractive strategy for cancer treatment, and ovarian cancer is an ideal target for immunotherapies because these tumors are generally immunogenic, providing tumor-associated antigens. Considering bacteriophage virus-like nanoparticles (QβVLPs) are an attractive tool for stimulating immune responses, Wang et al. developed biocompatible and biodegradable QβVLPs-loaded Mg-based micromotors to treat ovarian cancer (Wang et al., 2020) (Figure 4B). The Qβ-motors are tested *in vitro* (mouse intraperitoneal fluid) to have an average speed of ∼60 μm/s. Besides, they demonstrated that Q-motors actively deliver intact immunostimulatory QβVLPs, greatly enhancing the distribution and retention time of the QβVLP payload in the tumor microenvironment, leading to tumor growth suppression in the peritoneal cavity. Wavhale and his team reported an innovative approach to performing tumor treatment using Mg-based microrobots with surface modification [anti-EpCAM antibody/transferrin, cyanine 5 NHS (Cy5) dye, fourth generation (G4) dendrimers, and glutathione] by chemical conjugation (Wavhale et al., 2021). The Mg-based Janus microrobots can perform autonomous motion in PBS, DMEM, or serum with speeds of 0.815 ± 0.086 mm/s, 0.707 ± 0.06 mm/s, and 0.393 ± 0.07 mm/s, respectively, and can realize tumor cell capture (Figure 4C). With real-time imaging technology and precise magnetic guidance, we can envision that captured tumor cells can be transported outside the human body.

Gastric cancer is common and frequently causes cancer-related death. Significantly advanced gastric cancer with serosal invasion is usually undetectable. Nevertheless, researchers have shown that delivering anti-cancer drugs *via* MNRs is a feasible way to safely treat gastric cancer (Nakamura et al., 2005). Zhou et al. (2019) for example, created poly (amino acid)-based microrockets that can be propelled by gastric acid and navigated by a magnetic field (Figure 4D). All the materials used for constructing microrockets [i.e., poly (aspartic acid), Zn, Fe] can be decomposed by gastric acid or protease in the digestive tract (Thombre and Sarwade, 2005). Furthermore, various side-chain functional groups (e.g., carboxyl, sulfhydryl, hydroxyl, amino) on the poly (amino acid)s can be modified, allowing PASP/Fe-Zn microrockets to serve as microcarriers *via* multiple intermolecular interactions for a variety of bioapplications. The motion speed of the real-time magnetically guided PASP/Fe−Zn microrockets in the gastric acid simulant is measured as 29.2 ± 7.9 μm/s.

Besides active metals, carbonate can also be used to design MNRs powered by biological media. Guix et al. (2016) have demonstrated the feasibility of using biocompatible calcium carbonate Janus motors moving without any surfactants and external fuel additions but with the presence of fuel created *in situ* by HeLa cells (Figure 4E). Calcium carbonate micromotors are synthesized under sonication mixing conditions and conveniently dispersed onto glass substrates to deposit a 0.8 nm cobalt layer. These Janus motors show a continuous and directional motion with a speed of 0.544 μm/s in the Petri dish with harbored HeLa cells (pH ≈ 6.5).

Inspired by endogenous biochemical reactions in the human body involving the conversion of amino acid L-arginine to nitric oxide by NO synthase or reactive oxygen species (ROS), Wan et al. have reported a nanomotor made of hyperbranched polyamide/L-arginine (HLA) (Wan et al., 2019). L-arginine not only provides fuel for the production of NO that generates driving force but also provides beneficial effects, including promoting endothelialization and anticancer effects, along with other valuable by-products like L-citrulline that can improve immune system function, maintain joint function, balance normal blood sugar levels, and contain rich antioxidants that absorb harmful free radicals (Figure 4F) (Lutgens et al., 2003; Fan et al., 2017). Furthermore, the self-imaging of the nanomotors in the cellular condition is realized with the help of the suitable fluorescent property of HPAM, providing the possibility of tracking the devices *in vivo* in the future. The speed was measured *in vitro* and proved to increase with the concentration of the L-arginine during the synthesis process (HLA_10_, HLA_15_, and HLA_20_ ≈ 3, 8, and 13 μm/s). Taking account of the over-expression of FA receptors on cancer cells, they adopt an FA-mediated targeting delivery strategy and produce HFLA_10_ nanoparticles (heparin/FA/L-arginine). Co-culturing HLA_10_ and HFLA_10_ with MCF-7 cells demonstrated an enhanced cellular uptake by FA-modification. Their work presents a zero-waste, self-destroyed, and self-imaging nanomotor with the potential biological application for the treatment of various diseases in different tissues, including blood vessels and tumors.

Owing to the biocompatibility and self-propelling ability, researchers confirmed the feasibility of applying biocompatible fuel-powered MNRs to drug delivery and detoxification under different circumstances (i.e., cell culture, biofluids, and living mice) in many preclinical experiments. Although biocompatible fuels may reduce the undesirable effects in the physiological environment compared to toxic fuels, the accumulation of their associated waste products may exert an undesired effect on healthy tissues. Attempts have been made toward biocompatible fuel-powered MNRs that will not produce harmful waste, such as the above-mentioned human leukocyte antigen (HLA) nanomotor. Furthermore, various biocompatible fuels may provide MNRs with a powerful driving force, and thus more effort needs to be put into exploration.

#### 4.1.3 Enzyme-powered micro/nanorobots

Enzyme-powered MNRs rely on biocatalytic reactions of widely available biocompatible fuel substrates. Therefore, they can also be powered by biocompatible bioavailable fuels, such as urease, glucose, and lipase, and are considered to be promising drug-delivery platforms for tumor therapy.

**FIGURE 5:**
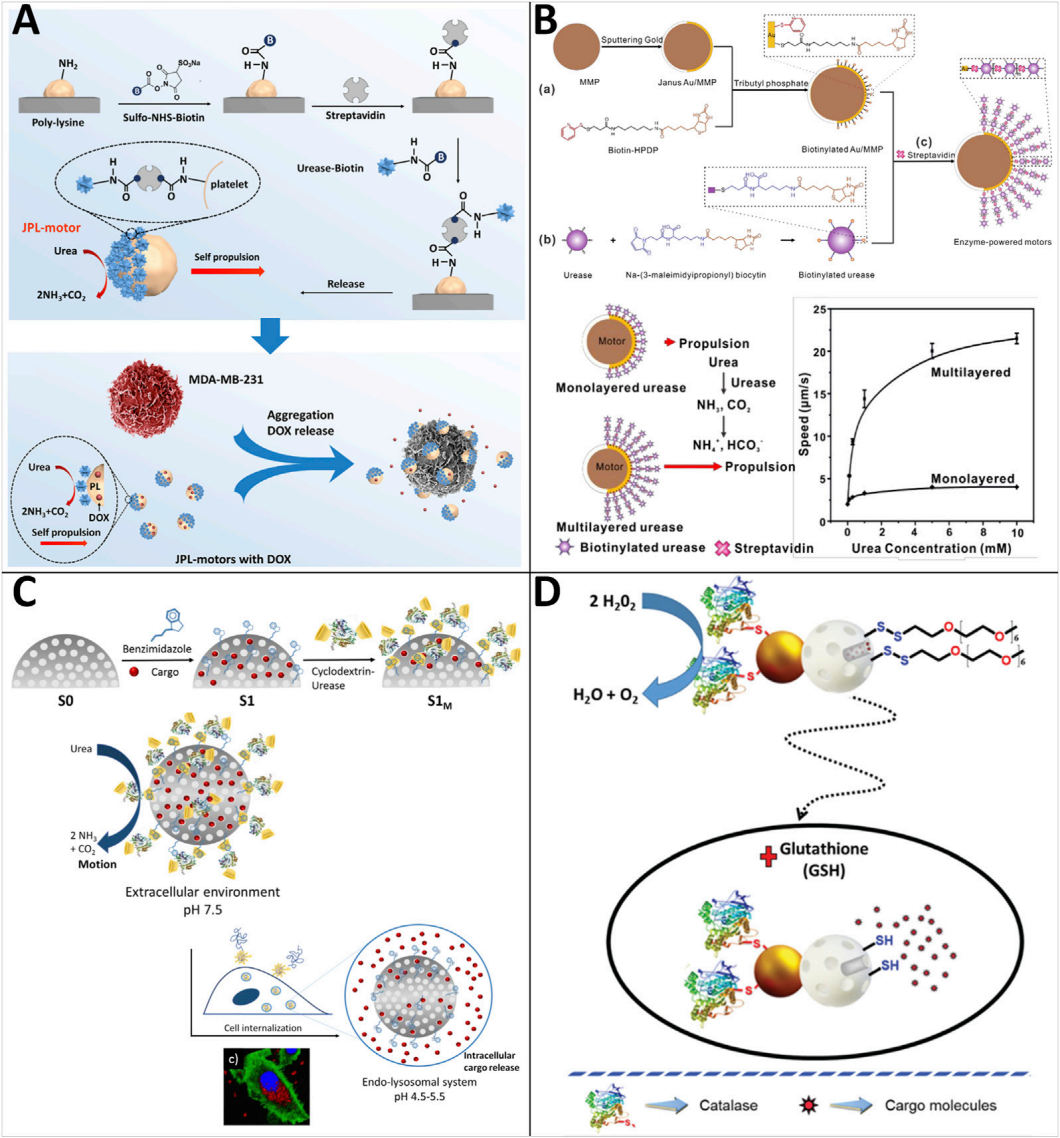
Motile drug delivery by enzyme-powered MNRs. **(A)** Enzyme-powered Janus platelet micromotor. (Tang et al., 2020) Copyright 2020. Reproduced with permission from American Association for the Advancement of Science. **(B)** Multilayer-urea-based Janus Au/magnetic microparticles. (Luo et al., 2020) Copyright 2020. Reproduced with permission from American Chemical Society. **(C)** Enzyme-powered gated mesoporous silica nanomotors. (Llopis-Lorente et al., 2019b) Copyright 2019. Reproduced with permission from American Chemical Society. **(D)** Enzyme-powered Janus Au-mesoporous silica nanoparticles. (Llopis-Lorente et al., 2019a) Copyright 2019. Reproduced with permission from The Royal Society of Chemistry.

Platelets play an essential role in the human body and can target particular objectives (e.g., cancer cells, bacteria) *via* their receptors expressed on the surface (Menter et al., 2014; Garraud, 2015). Integrating platelets and synthetic materials to produce cell-based MNRs is a novel attempt. Therefore, Tang et al. (2020) presented an endogenous enzyme-powered Janus platelet micromotor (JPL-motor) system prepared by immobilizing urease asymmetrically onto the surface of natural platelet cells (Figure 5A). The JPL-motor inherits the bio-functionalities of the platelets, such as practical targeting ability towards cancer cells and bacteria. The Janus distribution of urease over the platelet surface results in the asymmetric biocatalytic decomposition of urea into ammonia and carbon dioxide. Subsequently, a concentration gradient and active directional flow of reaction products around JPL-motors are generated, which drives the JPL-motor to undergo self-diffusiophoretic propulsion. They demonstrated that the JPL-motor could achieve self-propulsion in the presence of urease, specifically target cancer cells and bacteria, and improve the anti-cancer and bacteria efficacy.

To solve the problem of the weak driving force that limits the biomedical application of enzyme-powered MNRs, Luo and coworkers developed an approach to significantly enhance the propulsion of enzyme-powered micromotors by the multilayered assembly of enzymes (Luo et al., 2020) (Figure 5B). The urease-powered micromotors consist of multilayered ureases asymmetrically immobilized on biotinylated Janus Au/magnetic microparticles through the assembly of biotinylated ureases using streptavidins as cross-linkers. The magnetic microparticles enable fast magnetic separation and the guidance of the external magnetic field. They showed that micromotors could be self-propelled with an average speed up to about 21.5 ± 0.8 μm/s at physiological urea concentrations (10 mM). Their notable work makes it a step further toward the drug delivery application of enzyme-powered MNRs.

Llopis-Lorente and coworkers reported enzyme-powered gated mesoporous silica nanomotors for on-command intracellular payload delivery (Figure 5C) (Llopis-Lorente et al., 2019b). The nanomotors are made up of mesoporous silica nanoparticles that have been functionalized with benzimidazole groups, loaded with a dye {e.g., [Ru (bpy)_3_]Cl_2_} or a model drug (e.g., DOX), and capped with cyclodextrin-modified urease (CD-U) through the formation of inclusion complexes between CD-U and the benzimidazole. Cargo release is triggered when nanomotors enter a low pH environment (e.g., tumor tissue) as protonation of benzimidazole groups and dethreading of the supramolecular ensemble. In contrast, at physiological pH, the supramolecular benzimidazole:CD-U nanovalve can act as a bulky stopper and prevents cargo release. Furthermore, due to the acidity of lysosomal compartments, their studies with HeLa cells show that the presence of urea increases nanoparticle internalization and intracellular release of both [Ru (bpy)_3_]Cl_2_ and DOX (Figure 5D).

Most enzyme-powered MNRs take advantage of bio-friendly fuels, such as urea, glucose, and lipid. Functional enzyme-powered MNRs can respond to the environment or external signals to realize enhanced drug delivery and controlled drug release. Hence, multiple experiments have been carried out *in vitro* or *ex vivo* and have verified their ability to treat the tumor. Moreover, researchers have also successfully prolonged their lifetime by introducing bio-compatible components or surface functionalization. Despite the considerable progress, the enzyme-powered MNRs still have some important limitations, such as weak driving forces, high cost, difficult preservation, and deficient stability. Nanozyme is a series of artificial nanomaterials that can simulate biological enzyme activity. Compared with natural enzymes, nanozymes can achieve low-cost and large-scale production and maintain stability in non-physiological environments for a long time (Wang et al., 2022). Therefore, it can be used as an alternative to power MNRs (Mujtaba et al., 2021).

It is worth noting that even state-of-the-art chemically-powered MNRs, whether made of polymers, metals, oxides, or enzymes, are seldom applicable for operations involving blood flow and the intracellular environment, as they have some inherent limitations. Chemically-powered MNRs are typically very weak (velocities on the order of tens of lengths per second or less) under biologically relevant conditions and are difficult to control (speed, directionality, etc.). Nonetheless, researchers will continue to find ways to facilitate their chemical propulsion in biological fluids and target the tumor site for efficient anti-tumor treatments.

### 4.2 Motile drug delivery by external-field-powered micro/nanorobots

Those MNRs powered by external physical fields (such as ultrasound, magnetic, and light fields) do not require chemical fuels like chemically-propelled MNRs, and also show many additional advantages like easy actuation, precise control, and strong propulsion. Thus, they are widely employed to perform drug delivery tasks. The representative examples are summarized in Table 2.

**TABLE 2:** External-field-powered MNRs for motile-targeting drug delivery.

Energy sources		Representative examples	Environment	Motion behavior	Loaded cargo/therapeutic drugs	Biomedical application	Ref.
Light	NIR	Pt NPs modified polymer multilayer micromotors	*In vitro*	Maximum speed ≈62 μm/s	N.A.	Photothermal therapy	Wu et al. (2014)
		MPCM@JMSNMs	*In vitro*	0.9 μm/s ∼ 5.98 μm/s	Propidium iodide	Drug delivery for cancer	Xuan et al. (2018)
		Membrane-cloaked Janus polymeric motors	*In vitro*	2.33 μm/s ∼ 19.8 μm/s	Heparin	Drug delivery and Photothermal therapy for thrombus	Shao et al. (2018)
		Photothermally-driven polymersome nanomotors	*In vitro*	≈1.9 μm/s ∼ 6.2 μm/s	Propidium iodide	Intracellular Drug delivery	Shao et al. (2020)
		Platelet-derived porous nanomotors	*In vitro*	Maximum speed ≈4.5 μm/s	Urokinase	Thrombus therapy	Wan et al. (2020)
			*In vivo*		Heparin		
		Janus calcium carbonate particle micromotors	*In vitro*	2.9 μm/s ∼ 7.3 μm/s	DOX	Drug delivery	Zhou et al. (2021)
	X-ray	Half-copper-coated silica (Cu/SiO_2_) Janus microparticles	*In vitro*	Maximum speed ≈1.2 μm/s	N.A.	Potential for enhancing diagnosis and radiotherapy	Xu Z et al. (2019)
	UV	Photoelectrochemical TiO_2_-Au-nanowire-based motors	*In vitro*	Speed of 5.6 ± 1.5 μm/s	N.A.	Ocular disease (neural RGC stimulation)	Chen et al. (2021)
Ultrasound		Asparaginase-modified nanowire motors	*In vitro*	5 μm/s ∼ 60 μm/s	Asparaginase	Cancer cells inhibition	Uygun et al. (2017)
		Cas9-sgRNA@AuNW motors	*In vitro*	≈22 μm/s	Cas9-sgRNA Complex	Drug delivery (e.g., gene therapy)	Hansen-Bruhn et al. (2018)
		Liquid metal nanomachines	*In vitro*	4.6 μm, 420 kHz, 47.4 μm/s	N.A.	Photothermal therapy for cancer	Wang et al. (2018)
		AuNS functionalized polymer multilayer tubular nanoswimmers	*In vitro*	5 μm/s ∼ 80 μm/s	N.A.	Potential for various biomedical applications (e.g., gene delivery)	Wang Q et al. (2019)
		RBCM-micromotors	*In vitro*	Maximum speed ≈56.5 μm/s	Oxygen and ICG	Photodynamic cancer therapy	Gao et al. (2019)
Magnetic field		Multifunctional nanorobot systems (MF-NRS)	*In vitro*	4.5 ± 2.2–10.37 ± 5.3 mm/s	DOX	Chemo-phototherapy for cancer	Jin et al. (2019)
			*In vivo*				
		HADMSC-based medical microrobots	*In vitro*	N.A.	Mesenchymal stem cell	Cartilage repair	Go et al. (2020)
			*In vivo*				
		Bilayer hydrogel sheet-type intraocular microrobots	*In vitro*	N.A.	DOX	Ocular disease (e.g., retinoblastoma)	Kim et al. (2020)
		Leukocyte-inspired mult-ifunctional microrollers	*In vitro*	600 μm/s	DOX	Various diseases (e.g., cancer)	Alapan et al. (2020)
		Photosynthetic bohybrid nanoswimmers	*In vitro*	Maximum speed ≈78.3 μm/s	Chlorophyll	Cancer treatment	Zhong et al. (2020)
			*In vivo*				
		Sequential magneto-actuated and optics-triggered biomicrorobots	*In vitro*	Average speed: 13.3 ± 4.5 μm/s	ICG nanoparticles	Various disease (e.g., cancer)	Xing et al. (2021)
			*In vivo*				
		ICG/R837 loading and DPA-PEG coating magnetic nanoparticles	*In vivo*	N.A.	ICG and immune-ostimulator R837 hydrochloride	Photothermal/immunotherapy for cancer	Zhang et al. (2020)
		Magnetic tri-bead microrobots	*In vitro*	Average velocity: 14.5 μm/s	DOX	Photothermal therapy and chemotherapy	Song et al. (2021)
		Magnetic-actuated “capillary container”	*In vitro*	N.A.	N.A.	Selective fluid colle-ction, drug delivery	Zhang Y et al. (2021)
		Personalized magnetic micromachines	*In vitro*	Maximum speed: 9.3–9.8 μm/s	Cell tracker deep red dye	Potential for drug delivery	Ceylan et al. (2021)
		Au-Ni nanowires	*In vitro*	6.35–21.5 μm/s	DOX and ssDNA	Drug delivery	Karaca et al. (2021)
		Nickel-based spherical Janus magnetic microrobots	*In vitro*	0.97 ± 0.27 mm/s	N.A.	Potential for drug delivery	Wrede et al. (2022)

#### 4.2.1 Ultrasound-powered micro/nanorobots

**FIGURE 6:**
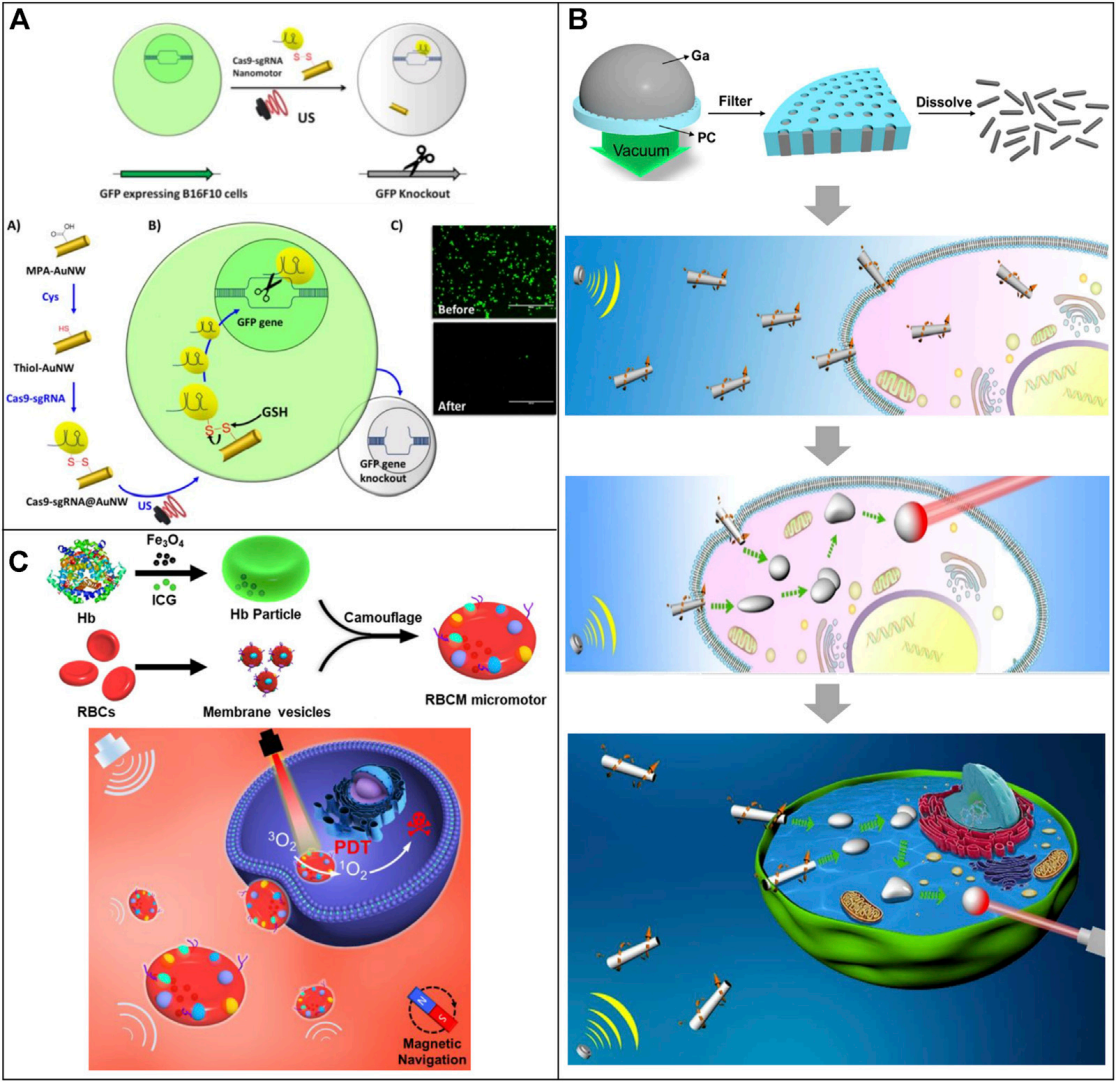
Motile drug delivery by ultrasound-powered MNRs. **(A)** Red blood cell mimicking-micromotor. (Hansen-Bruhn et al., 2018) Copyright 2018. Reproduced with permission from John Wiley and Sons. **(B)** Shape-transformable, fusible rodlike swimming liquid metal nanomachine. (Wang et al., 2018) Copyright 2018. Reproduced with permission from American Chemical Society. **(C)** Cas9-sgRNA@gold nanowire (AuNW) motor. (Gao et al., 2019) Copyright 2019. Reproduced with permission from American Chemical Society.

Due to their biocompatibility, low-power ultrasonic waves have been widely used in clinical applications, so the research of nanomotors using ultrasound as propulsion has received considerable attention. Ultrasound-powered MNRs require no fuel and can change speed by adjusting the applied power. Hansen-Bruhn and coworkers proposed a novel application of ultrasound-powered MNRs for rapid and efficient intracellular delivery of functional Cas9-sgRNA complexes using the knockout of the GFP encoding gene as a model (Hansen-Bruhn et al., 2018) (Figure 6A). The Cas9-sgRNA@gold nanowire (AuNW) motor was fabricated by immobilizing the Cas9-sgRNA complex onto the surface of thiol functionalized AuNWs through a reversible disulfide linkage *via* cysteine residues within Cas9. These nanomotors could internalize into GFP-expressing B16F10 cells and dramatically decrease the fluorescence intensity through knockout of the GFP encoding gene after GSH-mediated release of Cas9-sgRNA from the nanomotors. They also confirmed that these Cas9-sgRNA-loaded nanomotors are capable of rapidly penetrating cell membranes and markedly improving the speed and efficiency of the gene knockout process. In addition, further surface modification, such as integrating with a specific receptor (e.g., FA receptor, HER-2 receptor) or antibodies, can facilitate targeting drug delivery toward the tumor.

Wang et al. developed a shape-transformable and fusible rodlike swimming liquid metal nanomachine based on the biocompatible and transformable liquid metal gallium. They can be propelled by an ultrasound-induced primary acoustic radiation force in the levitation plane at a controllable speed (Wang et al., 2018) (Figure 6B). A pressure-filter-template technology was used to prepare these MNRs, and the diameter and length could be controlled by adjusting the nanoporous templates, filter time, and pressure. The resulting propulsion force by acoustic energy can actively pierce and internalize liquid metal MNRs into HeLa cells. The liquid metal nanomachine can actively seek cancer cells and transform from a rod to a droplet after drilling into cells owing to the removal of gallium oxide layers in the acidic endosomes. These transformed MNRs could fuse inside cells and photothermally kill cancer cells under the illumination of near-infrared light.

Gao and coworkers developed an acoustic-powered, magnetic navigable red blood cell-mimicking (RBCM) micromotor to transport oxygen and photosensitizers to enhance the photodynamic therapeutic effect (Gao et al., 2019) (Figure 6C). The RBCM micromotors consist of biconcave RBC-shaped magnetic hemoglobin cores encapsulating photosensitizers and natural RBC membrane shells. External ultrasonic and magnetic fields can precisely control the speed and direction of the RBCM micromotors. Moreover, due to the RBC membrane coating, the RBCM micromotors possess anti-biofouling capability and immune-clearance escape capability that help them prolong the motion time in blood. Upon irradiation of 808 nm light, the generated photodynamic effect of RBCM micromotors could rapidly kill cancer cells with an enhanced photodynamic cancer therapy efficacy.

Ultrasound, as a biocompatible, contact-free, and non-invasive energy source, can propel and manipulate the motion of MNRs. Moreover, it can provide MNRs with adequate propulsion in highly viscous and high-ionic bio-media. The existing ultrasound-powered MNRs display efficient and controllable movement in bio-media and perform on-demand tasks such as intracellular enzyme delivery, cell penetration, targeting gene knockout, and targeting photodynamic therapy. However, they usually suffer from poor control in motion directions and a limited transport space of their operating setups.

#### 4.2.2 Magnetic-field-powered micro/nanorobots

Magnetic-field-powered MNRs overcome most of the shortcomings presented by other propulsion principles, such as poor biocompatibility of chemical propulsion, lack of directional control of ultrasonic actuation, etc. They are actively studied as they are expected to enhance the targeting capabilities of micro/nanoscale systems (e.g., drug delivery systems).

**FIGURE 7:**
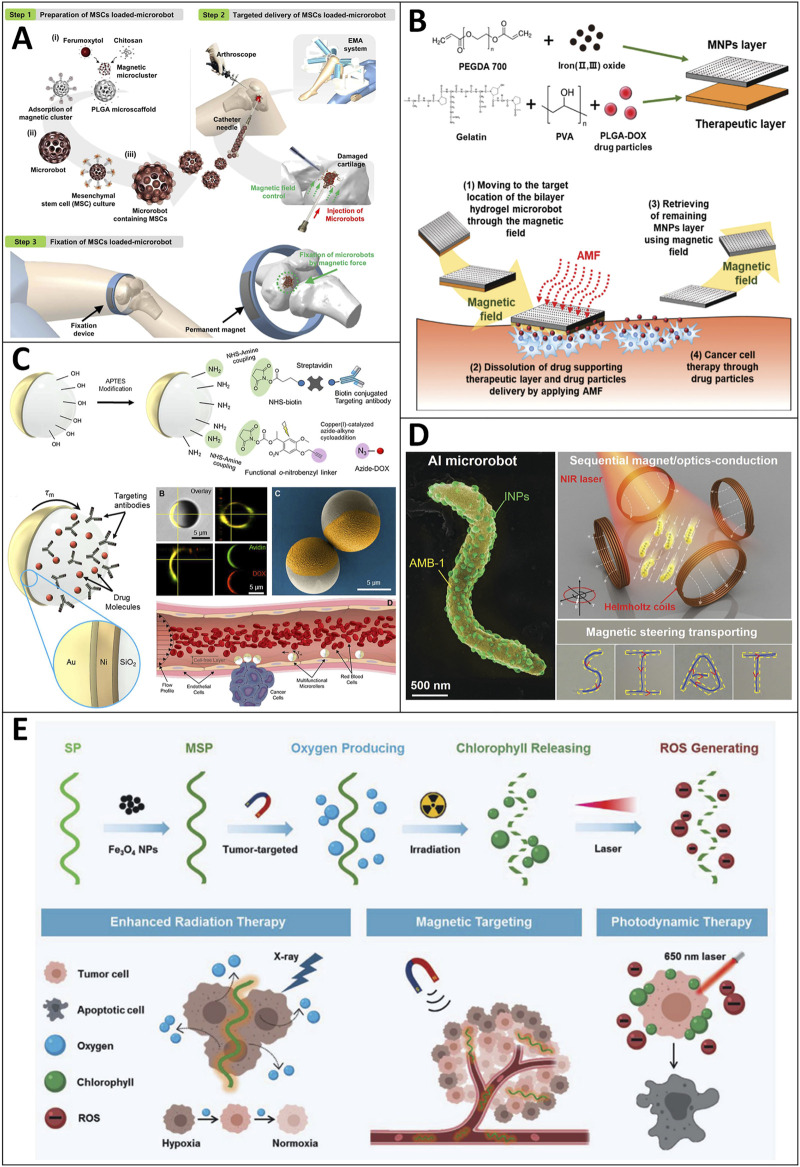
Motile drug delivery by magnetic-field-powered MNRs. **(A)** Human adipose-derived mesenchymal stem cell (hADMSC)-based medical microrobot. (Go et al., 2020) Copyright 2020. Reproduced with permission from American Association for the Advancement of Science. **(B)** Bilayer hydrogel sheet-type intraocular microrobots. (Kim et al., 2020) Copyright 2020. Reproduced with permission from John Wiley and Sons. **(C)** Leukocyte-inspired multifunctional microrollers. (Alapan et al., 2020) Copyright 2020. Reproduced with permission from American Association for the Advancement of Science. **(D)** Sequential magneto-actuated and optics-triggered biomicrorobots. (Xing et al., 2021) Copyright 2021. Reproduced with permission from John Wiley and Sons. **(E)** Photosynthetic biohybrid nanoswimmers. (Zhong et al., 2020) Copyright 2021. Reproduced with permission from John Wiley and Sons.

Mesenchymal stem cells (MSCs) are used in cartilage repair due to their notable advantages because they can induce peripheral tolerance and migrate to injured tissues, prevent arthritis by relieving inflammation, and treat joint cartilage through chondrogenic differentiation to avoid or delay joint replacement surgery (Mobasheri et al., 2014). Due to the low targeting efficiency of MSCs, the current MSC-based therapy requires a large number of cells for intra-articular injection or invasive surgery for scaffold implantation above millimeter size. Therefore, Go and coworkers proposed a human adipose-derived MSC (hADMSC)-based medical microrobot system that can realize targeted MSC delivery while containing minimally invasive procedures by needle-based injection, targeting, and fixation of the microrobot (Go et al., 2020) (Figure 7A). The microrobot system consists of a magnetic microrobot body capable of supporting MSCs, an electromagnetic actuation (EMA) system for 3D microrobots targeting the damaged cartilage, and a magnet for fixation of the microrobot. The experiments confirmed the feasibility of this system in cartilage repair *in vitro* and *in vivo*.

Ocular diseases such as retinoblastoma are challenging to cure using a drug delivery strategy due to the eye barrier and potential side effects (e.g., conjunctivitis, intraocular inflammation, cataract, etc.). Hence, a drug delivery system that can precisely control drug delivery is highly desirable. Kim et al. proposed a bilayer hydrogel microrobot for drug delivery capable of minimizing eye invasion, accurately delivering the drug to the ocular lesion, and retrieving the remaining part after drug delivery (Kim et al., 2020) (Figure 7B). After the therapeutic layers (i.e., a therapeutic layer of gelatin/PVA and PLGA-DOX) are dissolved, the loaded drugs are released around the lesion, and the remaining magnetic nanoparticle layer is retrieved using a magnetic field. The experiments were conducted in Roswell Park Memorial Institute medium cultured with retinoblastoma Y79 cancer cells, and the results confirmed that the therapeutic layer of the bilayer hydrogel microrobot has therapeutic effects on retinoblastoma Y79 cancer cells. In addition, the targeting and retrieval ability of the bilayer hydrogel MNRs are promising for treating eye diseases such as retinoblastoma.

The circulatory system appears to be an ideal route for the drug delivery of MNRs. The dynamic flow in the blood vessels hinders the propulsion of these drug carriers (Abbott et al., 2007; Soler et al., 2013). Considering the decreased flow velocities and a cell-free layer near the vessel walls, surface-enabled propulsion of MNRs (e.g., rolling or crawling) would be more efficient and robust. Hereby, Yunus Alapan et al. designed the leukocyte-inspired multifunctional microrollers (3.0 and 7.8 μm in diameter), which are composed of spherical Janus microparticles with a magnetically responsive half-side and a silica half-side for biochemical functionalities (i.e., anti-body HER2) and cargo loading (i.e., DOX) (Alapan et al., 2020) (Figure 7C). Different targeting moieties and drugs can be adopted according to cancer types or other diseases’ features. They showed that the multifunctional microrollers could be actuated and steered by external rotating magnetic fields, reaching a translational speed of 600 μm/s (corresponding to 76 body lengths per second) for drug delivery.

Xing et al. reported a sequential magneto-actuated and optics-triggered biomicrorobot (AI microrobot) for actively targeted cancer treatment (Xing et al., 2021) (Figure 7D). The AI microrobot consists of two components. The first one is the magnetospirillum magneticum (AMB-1), which provides the ability to autonomously swim toward the tumor site *via* internal hypoxia-driven effects and an externally applied magnetic field. Besides, the applied magnetic field can also be used to navigate the AI microrobots to facilitate drug delivery. The other component is the indocyanine green nanoparticles, which serve as a fluorescence imaging agent and a photothermal-therapy agent. Thus, the distribution and location of AI micromotors could be tracked in real-time. When applied to NIR light, the indocyanine nanoparticles are activated to ablate cancer cells by photothermal therapy. The results also showed that the AI microrobots can sequentially migrate to the internal hypoxic area of tumors and then effectively eradicate solid tumors through photothermal treatment under NIR laser irradiation.

Microalgae like *Spirulina platensis* have drawn much attention. They have been widely studied due to their advantageous features, which are biomass abundance in the natural environment, the effective photosynthetic capacity of oxygen production, inherent autofluorescent pigments, and so on (Moreno-Garrido, 2008; El-Gamal, 2010). Therefore, Zhong and coworkers proposed a multifunctional theranostic agent by integrating *Spirulina platensis* with low-cytotoxicity magnetite Fe_3_O_4_ NPs, which serves as a magnetic navigation and magnetic resonance imaging (MRI) contrast agent (Zhong et al., 2020) (Figure 7E). Furthermore, the inherent pigment (i.e., chlorophyll) not only serves as a photosensitizer for photodynamic therapy due to ROS generation upon 650 nm laser irradiation, but it also serves as a theranostic agent for fluorescence and photoacoustic imaging due to its autofluorescence property and absorbance peak at 680 nm (Pandey et al., 2006; Murchie and Lawson, 2013; Rizzi et al., 2016). Besides, the microswimmer can efficiently produce O_2_ to ameliorate tumor hypoxia and enhance radiotherapy efficacy. Their cancer cell-bearing mice experiments showed no noticeable weight changes, substantial organ damage, or inflammatory lesions. The tumor volume was significantly reduced by 98.7% *in vivo*. Thus, their team presented an ideal candidate for cancer diagnosis and multimodal therapy.

Magnetic-field-driven MNRs have drawn a lot of attention due to their versatility. They can be used in tumor targeting therapy and treat other diseases or environmental remediation. They can achieve highly controllable motion and move to inaccessible areas for drug delivery or targeted therapy. The magnetic component can serve as motion control and can be used as imaging contrast. However, it is notable that non-biocompatible magnetic elements cause potential risks. In future studies, it is imperative to find a safe and non-invasive way to remove them from the human body.

#### 4.2.3 Light-driven micro/nanorobots

Three types of light sources are used to propel MNRs, including ultraviolet, visible, and near-infrared light. Moreover, the light field can also be used to induce swarming behaviors, act as a motion switch, or drug release trigger. Under proper irradiation time, light-propelled MNRs can carry out the on-demand mission with minimal invasion. Additionally, these MNRs exhibit special abilities such as immunity, selective targeting, and tissue penetrating with surface modification.

##### 4.2.3.1 NIR light

**FIGURE 8:**
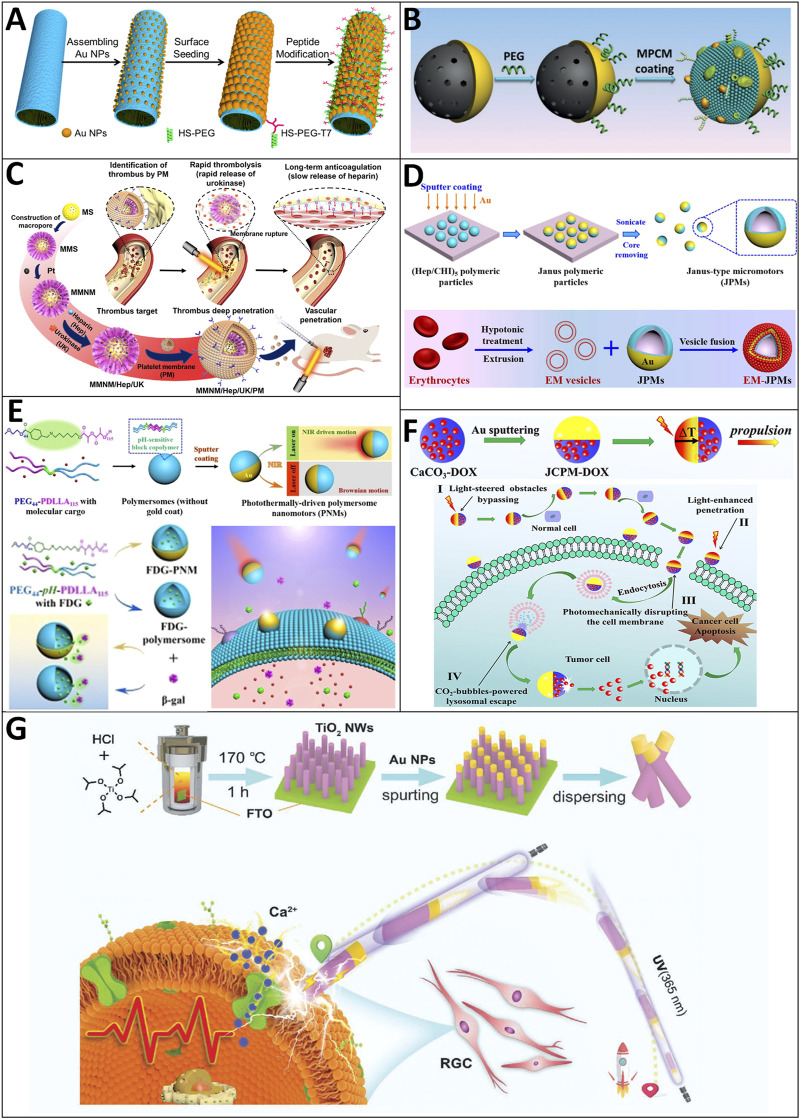
Motile drug delivery by light-driven MNRs. **(A)** Platinum nanoparticle (Pt NP)-modified polymer multilayer micromotors. (Wu et al., 2014) Copyright 2014. Reproduced with permission from American Chemical Society. **(B)** Macrophage cell membrane (MPCM)-camouflaged Janus mesoporous silica nanomotor (JMSNM). (Xuan et al., 2018) Copyright 2018. Reproduced with permission from John Wiley and Sons. **(C)** Platelet-derived porous nanomotors. (Wan et al., 2020) Copyright 2020. The Authors, some rights reserved; exclusive licensee American Association for the Advancement of Science. **(D)** Photothermally-driven polymersome nanomotors. (Shao et al., 2018) Copyright 2018. The Authors, some rights reserved; exclusive licensee American Chemical Society. **(E)** Membrane-cloaked Janus polymeric motors. (Shao et al., 2020) Copyright 2020. The Authors, some rights reserved; exclusive licensee John Wiley and Sons. **(F)** Janus calcium carbonate particle micromotors (JCPMs). (Zhou et al., 2021) Copyright 2021. Reproduced with permission from Elsevier Inc. **(G)** Photoelectrochemical TiO_2_-Au-nanowire-based motor. (Chen et al., 2021) Copyright 2021. Reproduced with permission from John Wiley and Sons.

Wu and coworkers demonstrated that the NIR-triggered “on/off” motion of platinum nanoparticles (PtNPs)-modified polymer multilayer micromotors coated with a thin gold nanoshell (AuNS) and a tumor-targeted peptide can be achieved at a critical concentration of a peroxide fuel (0.1%, v/v) (Wu et al., 2014) (Figure 8A). Moreover, they proved that the photothermal effect could alone activate the on-demand motion of these micromotors in the absence of the peroxide fuel. The template-assisted layer-by-layer assembly and subsequent deposition of PtNPs inside and a thin AuNS outside were used to create these polymer multilayer motors. To make these micromotors suitable for conducting selective and minimally invasive treatment of cancer, the AuNSs of these motors are modified with a mixed monolayer of a tumor-targeted peptide and an antifouling PEG. Their experiments on targeted recognition ability and killing of HeLa cells by the photothermal effects are also illustrated successfully. Xuan et al. reported a near-infrared (NIR)-light-powered Janus mesoporous silica nanomotor (JMSNM) cloaked with the macrophage cell membrane (MPCM) that is capable of actively seeking cancer cells and thermomechanically percolating cell membrane (Figure 8B). The MPCM coating enables the nanomotors to inherit the immunological capability of the mother macrophages for specific recognition and their anti-bioadhesion properties, which results in good mobility of the NIR-powered nanomotors in biological media. Illuminated by NIR light, the locally generated temperature gradient forms a self-thermophoretic force along the asymmetric structure of MPCM@JMSNMs to achieve a directional motion. In addition, the synergistic effect between self-propulsion and the MPCM coating enables these nanomotors to thermomechanically open pores in the cytomembranes of cancer cells for guest molecule injection. They also showed the speed in PBS, cell culture medium (CM), and fetal bovine serum (FBS) at average velocities of 5.98 μm/s, 2.42 μm/s, and 0.91 μm/s, respectively (Xuan et al., 2018).

Venous thrombus is a frequently occurred but life-threatening disease. However, existing remedies have many limitations, such as the low drug utilization caused by the lack of targeting ability of thrombolytic drugs (Kobayashi et al., 2014). To solve this problem with advanced MNRs, Wan et al. developed mesoporous/macroporous silica/platinum nanomotors with platelet membrane (PM) modification (MMNM/PM) for sequentially targeting the delivery of thrombolytic and anticoagulant drugs for thrombus treatment (Wan et al., 2020) (Figure 8C). The drug delivery process consists of three stages. In stage 1: Due to the functionalization of the PM, these nanomotors can realize effective aggregation in the thrombus site. In stage 2, the applied NIR light is absorbed by the unevenly distributed Pt nanoparticles and generates a non-uniform heat distribution. Driven through thermophoresis, they can move into the thrombus, thus increasing the penetration depth and retention ratio of MMNM/PM nanomotors at the thrombus site. Then, PM can rupture under near-infrared (NIR) irradiation to achieve desirable sequential drug release, including the rapid release of thrombolytic urokinase (3 h) and the slow release of anticoagulant heparin (>20 days). Their experimental results confirmed that the synergistic effect of targeting ability from PM and motion ability from nanomotors could notably enhance the thrombolysis effect in both static and dynamic thrombus and rat models.

The cell membranes are the biological barriers that hamper intracellular drug delivery. To overcome this biological barrier, Shao et al. adopt a cell membrane disruption strategy to facilitate drug delivery, which takes advantage of the external-stimuli-induced effects (e.g., photothermal effects) that can locally disrupt cell membranes (Figure 8D) (Shao et al., 2018). They also use biodegradable poly (ethylene glycol)-b-poly (d, l-lactide) (PEG-PDaLLA) block copolymers, with the two blocks linked by a pH-sensitive imine bond, to create nanoscopic polymersomes, which are then modified with a hemispherical gold nanocoating. Upon NIR light illumination, the as-result nanomotors undergo autonomous motion in the direction opposite the source of light and display a laser-power-dependent speed that ranges from 1.9 ± 0.25 μm/s to 6.2 ± 1.10 μm/s. These nanomotors can traverse biological membranes and penetrate tumor tissues (Figure 8E) (Shao et al., 2020).

Successive biological barriers, as one of the challenges, could hinder the application of tumor therapy. Zhou et al. recently reported that Janus calcium carbonate particle micromotors (JCPMs) could achieve precise drug (i.e., DOX) delivery *via* a multistage self-adapted strategy that combines NIR-light driven navigation, photothermal cytomembrane traversing, autonomous tumor penetration, and microenvironment triggered CO_2_-propulsion in a cascaded manner to overcome sequential biological barriers (Zhou et al., 2021) (Figure 8F). The notable mobile ability of JCPMs comes from the self-thermophoretic force and thermomechanical perforation of the cytomembrane by the photothermal effect and the CO_2_ generation of CaCO_3_-based JCPMs under an acidic lysosomal microenvironment. The facilely fabricated biocompatible JCPMs with drug-specific-release capability exhibit potential for transporting multiple kinds of drugs.

##### 4.2.3.2 Ultraviolet light

Neuronal retinal ganglion cells (RGCs) are crucial in converting light into neural signals. Thus, failing to activate RGCs can lead to irreversible visual impairment or even more severe situations like blindness (Masland, 2012; Pearson et al., 2012; Packer et al., 2013). Existing treatments such as surgically implanting artificial photoreceptors and optogenetics either cause an invasive problem or are challenging. Chen and coworkers developed a minimally invasive and highly controllable approach to achieve RGC stimulation (Chen et al., 2021). In their work, the propulsion of the TiO_2_-Au nanowire-based motor takes advantage of UV-induced self-electrophoresis. It can be steered by adjusting the light direction to reach the RGCs with high precision (Figure 8G). They showed that these nanomotors display efficient propulsion i.e., a speed of 5.6 ± 1.5 μm/s in deionized water (pH = 7.1) when applied to an extremely low-intensity UV light field (50 mW/cm^2^). Once they reached the RGCs, the locally generated electric signals were transmitted to the targeted neuronal RGC, induced calcium response, and eventually triggered cell activation. Accordingly, the photoelectricity, calcium response, and cell activation state can be precisely adjusted by tuning the UV intensity. To the best of our knowledge, for the first time, their team demonstrated that the photoelectric conversion capabilities of MNRs can not only provide propulsion and be utilized for precise modulation of neuronal activities.

##### 4.2.3.3 X-ray

Conventional UV−vis/NIR light sources hold insufficient penetrating power deep into the biological medium, limited to a few millimeters (Ash et al., 2017). However, X-ray has been used in medical imaging and radiotherapy due to its powerful whole-body penetrating ability. Xu et al. first employed X-ray as the power source to generate propulsion for micromotors in an aqueous medium (Xu et al., 2019). The propulsion mechanism is based on the radiolysis of water, in which the particle motion follows the growth of the H_2_ bubble under X-ray irradiation. They showed that the instantaneous speed could be controlled by the variation of radiation dose. Although further effort must be put into studying the radiation-related safety problems, motors’ functionalization, etc., their micromotors hold the potential for diagnosis and radiotherapy in the future.

Even though external-field-powered MNRs have been widely used in tumor-target drug delivery, there are still some limitations in their future practical applications. For instance, ultrasound-powered MNRs are biocompatible but lack directional control. Light-driven MNRs may need H_2_O_2_ and high-intensity light sources, which may increase their toxicity. In contrast, magnetic-field-powered MNRs can overcome most of the shortcomings of other propulsion principles and are expected to be combined with MRI systems to enable imaging and tracking of MNRs and their propulsion and motion control.

### 4.3 Motile drug delivery by biohybrid micro/nanorobots

Biohybrid MNRs usually integrate synthetic nanoparticles or nanostructures with living cells (e.g., sperm, neutrophils, macrophages, etc.) or micro-organisms (e.g., erythrocytes, microalgae, *E. coli*, etc.) controlled by external or local stimuli to achieve precisely targeted tumor therapy. The biological section of the biohybrid MNRs employs an actuator and a sensor (Wang et al., 2022). Meanwhile, their artificial section offers support and other functions. Biohybrid MNRs have attracted significant attention due to their excellent biocompatibility, extremely low toxicity, high properties in drug protection, targeted transportation by special migration (e.g., chemotaxis and aerotaxis), and environmental-sensitive responses. The representative examples of biohybrid MNRs for motile drug delivery are summarized in Table 3.

**TABLE 3:** Biohybrid MNRs for motile-targeting drug delivery.

Energy sources		Representative examples	Experimental environment	Motion behavior	Loaded cargo/therapeutic drugs	Biomedical application	Ref.
Living cells		Sperm hybrid micromotors	*In vitro*	Average speed: 41 ± 10 μm/s	DOX hydrochloride	Drug delivery (e.g., cancer, diseases in the female reproductive system)	Xu et al. (2018)
		SHC hybrid sperm micromotors	*In vitro*	Average speed: 76 ± 17 μm/s in blood	Heparin	Diseases in the circulatory system (e.g., blood clot)	Xu et al. (2020a)
		Human-sperm-based hybrid micromotors	*In vitro*	N.A.	DOX and CPT	Gynecologic diseases (e.g., cervical cancer, ovarian cancer)	Xu et al. (2020b)
		Neutrobots	*In vitro*	Maximum speed: 16.4 μm/s	Paclitaxel	Drug delivery	Zhang H et al. (2021)
			*In vitro*				
Micro-organisms	Microalga	Microalga-powered biohybrid microswimmers	*In vitro*	Average speed: 156.13 ± 9.66 μm/s	FITC labeled dextran	Drug delivery	Yasa et al. (2018)
	*E. coli*	Soft erythrocyte-based bacterial microswimmers	*In vitro*	Average speed: 10.2 ± 3.5 μm/s	DOX and superparamagnetic iron oxide nanoparticle	Drug delivery	Alapan et al. (2018)
		TDNPP-coated *E. coli*	*In vitro*	N.A.	TDNPPs	Photodynamic cancer therapy Intracellular cargo (e.g., protein) delivery	Wu M et al. (2019)
		Engineered probiotics	*In vitro*	N.A.	PD-L1 and CTLA-4 antagonists	Immunotherapy	Canale et al. (2021)
			*In vivo*				
		Magnetic-sensing *E. coli*	*In vitro*	Average speed: 5µm/min ±2 μm/min	N.A.	Potential for drug delivery	Aubry et al. (2020)

#### 4.3.1 Sperm-based micro/nanorobots

Sperms have a variety of functional characteristics that aid in medical applications (e.g., drug delivery). First, they can generate powerful propulsion by beating sperm flagella, which makes them an ideal actuator for MNRs. Second, sperm have specific proteins on their membranes that can inhibit inflammation and proteasome suppressing immunoreactions. Most importantly, their ability to swim against blood flow (rheotaxis) and close to the boundaries (thigmotaxis) is beneficial for locomotion in the circulatory system (Miki and Clapham, 2013; Ishimoto and Gaffney, 2016).

**FIGURE 9:**
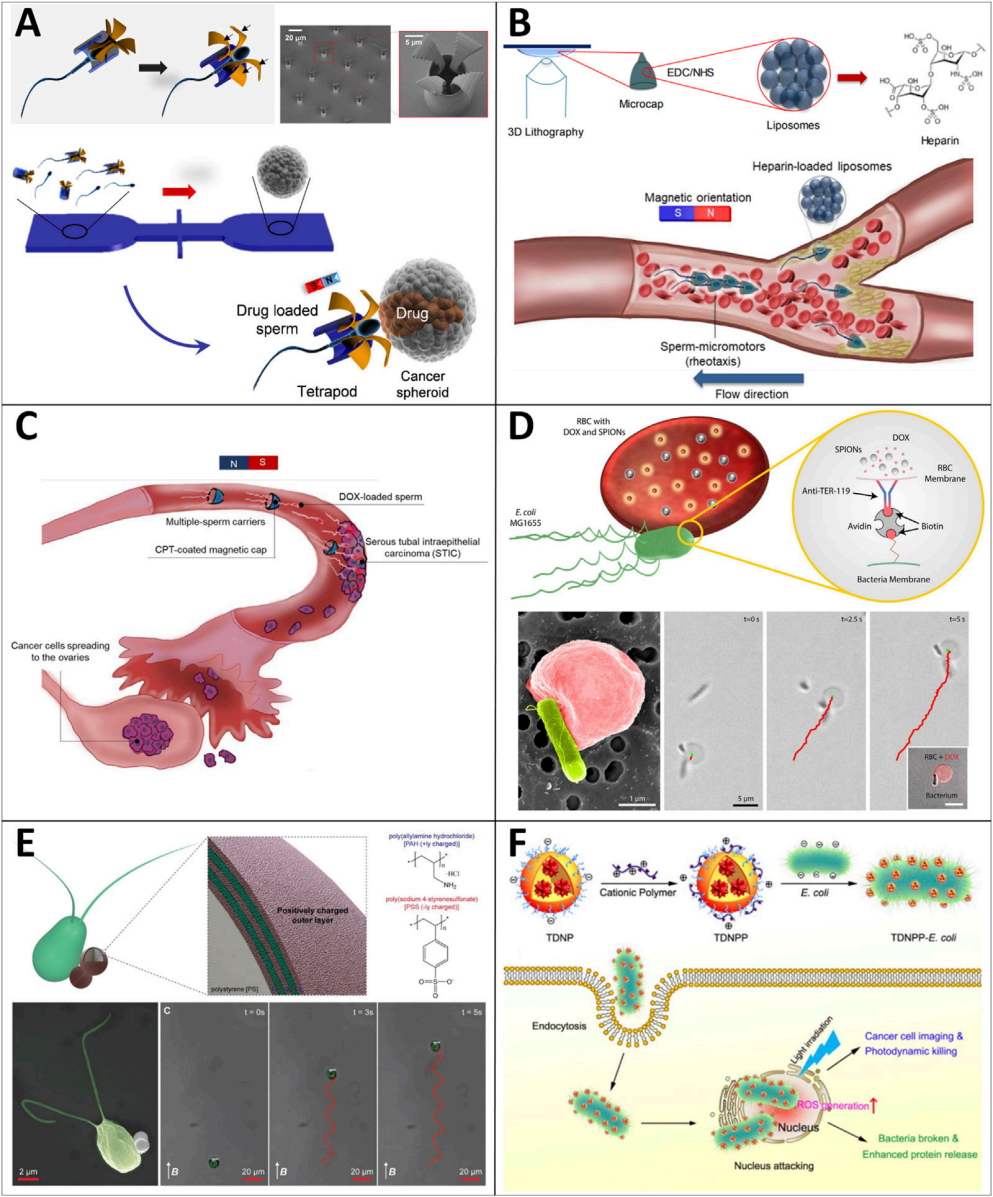
Motile drug delivery by biohybrid MNRs. **(A)** Sperm hybrid micromotors. (Xu et al., 2018) Copyright 2018. Reproduced with permission from American Chemical Society. **(B)** Streamlined-horned cap (SHC) hybrid sperm micromotors. (Xu et al., 2020a) Copyright 2020. Reproduced with permission from American Chemical Society. **(C)** Human-sperm based hybrid micromotors. (Xu et al., 2020b) Copyright 2020. Reproduced with permission from The Royal Society of Chemistry. **(D)** Soft erythrocyte-based bacterial microswimmers. (Alapan et al., 2018) Copyright 2018. Reproduced with permission from American Association for the Advancement of Science. **(E)** Microalga-powered biohybrid microswimmers. (Yasa et al., 2018) Copyright 2018. Reproduced with permission from John Wiley and Sons. **(F)** Poly-ethylenimine aggregation-induced emission photosensitizer nanoparticles (TDNPP)-coated *E. coli*. (Wu M et al., 2019) Copyright 2019. Reproduced with permission from American Chemical Society.

Xu et al. (2018) first developed the sperm-driven microrobot for drug delivery, which comprises a motile sperm cell that serves as the motile actuator and drug carrier, integrated with a 3D-printed magnetic 4-arm tubular microstructure (i.e., “tetrapod”) used for magnetic guidance and drug release (Figure 9A). The sperm-based MNR with a large drug capacity can efficiently swim in the physiological environment for a long time without causing a harmful immune response. Their experiment used bovine sperm as model cells to load DOX-HCl to treat cultured HeLa cells. The DOX-HCl was locally distributed into the spheroids, showing higher tumor cell-killing efficacy (87%) within the first 72 h compared to a simple drug solution (55%) with the same dose. Such sperm-hybrid MNRs have potential applicability for gynecologic cancer treatment and the therapy of other diseases in the female reproductive tract. Then, they presented streamlined-horned cap (SHC) hybrid sperm micromotors, which can efficiently and controllably swim against flowing blood (Xu et al., 2020a) (Figure 9B). These streamline-horned sperm micromotors can work as individuals or swarms to execute the task of heparin cargo delivery in flowing blood. These micromotors can carry out a controllable train-like assembly to further control the drug dose. Microcaps’ functionalization (e.g., antibodies, ligands, coagulation factors, equipment of stimuli-responsive materials) can make these micromotors capable of performing more complex tasks for biomedical applications.

Later, another example of the human-sperm-based hybrid MNRs was also reported by Xu and coworkers (Figure 9C) (Xu et al., 2020b). The micromotors display fast movement in the sperm medium by asymmetrically beating their flagellum, and the external magnetic field can guide them to achieve precise drug delivery when equipped with magnetic microcaps. They adopt a novel drug combination strategy by encapsulating DOX in the sperm nuclei and coupling hydrophobic drugs like CPT onto the microcaps. When the micromotors reach the target, the DOX can be efficiently taken up by cells, and hydrophobic drugs (e.g., camptothecin, CPT) function as complementary for sustained anti-cancer therapy due to their slow-release process. Besides, CPT can be changed into the active lactone form when existing in a relatively low pH range (tumor microenvironment pH 5–6). CPT’s pH-sensitive properties endow microcaps with tumor-selective ability, which improves the therapeutic effect on tumor tissue while reducing toxicity for normal cells nearby. In future studies, the MNRs can be functionalized with imaging agents, smart lysosomes, or polymers for further application.

#### 4.3.2 Bacteria-based micro/nanorobots

Although bacteria have been extensively used as actuators in the design of biohybrid MNRs, their possible acute toxicity and rapid growth in physiological environments limit their clinical application. Therefore, novel biohybrid MNRs with higher biocompatibility are highly desired for application in tumor-targeted therapy (Akolpoglu et al., 2022). Alapan and coworkers developed a multifunctional biohybrid micromotor composed of bioengineered bacteria (*E. coli MG1655*) and RBCs loaded with model drugs (i.e., DOX) and superparamagnetic iron oxide nanoparticles (Alapan et al., 2018) (Figure 9D). The RBC microswimmers can be propelled by the beating of bacteria flagella and steered by the external magnetic field, and the measured average velocity is 10.2 ± 3.5 μm/s. In addition, an on-demand NIR-activated hyperthermia termination switch is engineered for RBC MNRs to control the bacteria population after cargo delivery operations. Upon NIR light irradiation, indocyanine-green-loaded RBCs absorb the energy and convert it to heat, which results in a local temperature increment and kills the bacteria.

Another research led by Yasa presented a biocompatible biohybrid MNR powered by a unicellular freshwater green microalga (i.e., *Chlamydomonas reinhardtii*) (Yasa et al., 2018) (Figure 9E). Polyelectrolyte (PE)-functionalized magnetic spherical cargoes (1 µm in diameter) are attached to the surface of the microalgae *via* non-covalent interactions. The mobility test showed that the speed is relevant to the attaching position of the PE-functionalized PS microparticles on the microalgae, and the uniform-magnetic-field-steered microswimmers have a mean swimming speed of 51.44 ± 2.16 μm/s in the *x*-direction in 2D swimming analysis and a mean swimming speed of 156.13 ± 9.66 μm/s in 3D swimming analysis. Moreover, cytocompatibility of the microalgae and the biohybrid algal microswimmers is confirmed in cervical cancer (HeLa) cells, ovarian cancer (OVCAR-3) cells, and healthy cells (NIH 3T3). Furthermore, they verify the ability to deliver drugs by loading and transporting fluorescent isothiocyanate (FITC) labeled dextran to HeLa cells.

Recent research has reported that photosensitizers can damage bacteria membranes upon light irradiation and help achieve controllable drug release. Wu and coworkers developed biohybrid MNRs to achieve enhanced cancer cell imaging and effective photodynamic cancer therapy (Wu et al., 2019) (Figure 9F). The biohybrid system is composed of *E. coli* and the polymer matrix formed by photosensitizer nanoparticles (TDNPP) layer, which is fabricated by encapsulating aggregation-induced emission photosensitizers with a biocompatible block lipid-PEG copolymer to form TDNPP that are then coated by cationic polymer polyethyleneimine (PEI). The experiment showed that the bacteria-mediated photosensitizer delivery increases the protein release from *E. coli* and kills cancer cells by efficiently producing ROS upon light irradiation.

## 5 Conclusion and perspective

Researchers have devoted massive energy to moving forward with MNRs for the past two decades. Accordingly, there is a variety of MNRs with different advantageous characteristics that come to the fore. Despite bringing us a deeper understanding of propelling mechanisms, motion control, imaging technology, and so on, they still confront many challenges in “motile-targeting” drug delivery.

Compared with the conventional drug delivery system, chemically-powered MNRs can achieve self-propelled drug delivery. However, most of them are limited by their low biocompatibility, weak driving force, and short lifetime. Recent developments in external-field-propelled MNRs mainly utilize four external fields, including electric, ultrasound, light, and magnetic fields. Light-driven MNRs have many notable advantages; for example, they are easy to manipulate, fast to respond to light stimuli, and so on. Besides, near-infrared light also has the promising potential for controllable drug release and optical imaging (tracking the real-time location of MNRs) without causing any noticeable damage to the human body. Notwithstanding, the motion of the light-driven MNRs propelled by self-phoresis may be hampered by the high ionic environment in the human body. As for electrically-propelled MNRs, two non-negligible deficiencies preclude them from clinical applications—the local circuitry and enormous potential in the soft matter environment may lead to safety problems, and the electric field weakens rapidly with increasing distance. Because of their inherent advantages, ultrasound-driven and magnetically-propelled MNRs have received a lot of attention in recent decades. Ultrasound can provide MNRs with strong propulsion in biofluids with high viscosity and ionic strength. Furthermore, ultrasound can penetrate biological tissues deeply, almost without deleterious effects on biological systems, holding the potential for active drug delivery *in vivo*. Ultrasound-driven MNRs can be transported at a very high speed in aqueous solutions. Magnetically-propelled MNRs have the following advantages: First, a homogeneous magnetic field can be applied from a distance and holds a significant tissue penetrating ability. Second, the magnetic field can be used to image and control drug release. Third, under the actuation of the magnetic field, they can transport at a relatively high speed in biofluids. However, applying them to clinical trials has to overcome challenges of low intelligence (or poor autonomy) and the limited transport space of their operating setups. The above synthetic MNRs usually lack multifaceted biointerfacing capabilities and suffer from random agglomeration, non-specific bio-adhesion, cytotoxicity, and/or immune clearance when operating in *in-vivo* bioenvironments like blood vessels. Motile microorganism-based or cell-based biohybrid MNRs are promising candidates for drug delivery. Firstly, they have the potential to seek sites of disease because they inherit the actuating and sensing abilities of the microorganisms or cells. Secondly, they can take advantage of the nutrients in the environment and convert chemical energy into mechanical work to propel themselves. Thirdly, biohybrid microrobots can adapt themselves to the environment and have the ability to self-repair and self-assemble. Nevertheless, the biohybrid MNRs are still restrained by complex preparation, difficult preservation, and easy deactivation. Owing to the above challenges, many research attempts in “motile-targeting” drug delivery are limited to *in vitro* experiments, and only a few are demonstrated in animal models.

Future development of the MNRs for “motile-targeting” drug delivery should focus on their biological safety and functional efficacy *in vivo*. Regarding biological safety, biocompatible and biodegradable materials should be used to design and manufacture MNRs so that the synthesized MNRs do not cause organ damage and leave no residues after completing the drug delivery task. Secondly, proper biointerfacing strategies (e.g., polymer-brush grafting and cell-membrane cloaking) should be employed to engineer MNRs to enable them to simultaneously avoid random agglomeration, cytotoxicity, and immune clearance when working in *in-vivo* bioenvironments (Agrahari et al., 2020). In terms of the functional efficacy of MNRs *in vivo*, there are a few promising strategies or technologies that might facilitate drug delivery of MNRs. Firstly, nanozymes and single-atom catalysts catalyzing the conversion of endogenous chemicals (Lyu et al., 2020; Wu et al., 2020; Ding et al., 2021) should be employed to design chemically-powered MNRs owing to their superior performance in adjustability, stability, and easy synthesis compared to biological enzymes. Secondly, synthetic biology, as an emerging and converging scientific field, may bring new opportunities for biohybrid MNRs, including knocking out toxic genes, regulating and controlling the population of microorganisms, and even allowing microorganisms to synthesize drugs and deliver them to the target site (Danino et al., 2010; Danino et al., 2012; Liao et al., 2019). Thirdly, novel motile cell-based microrobots should be developed as they may simultaneously exhibit high biocompatibility, self-propelled ability, and intrinsic chemotaxis, and thus are envisioned to be less likely to be recognized by the immune system and deliver drugs in a self-targeting manner (Zhang et al., 2021). Fourth, although there are limited successful examples of the targeted drug delivery of swarming MNRs *in vivo*, swarming MNRs hold the potential to perform better in targeted drug delivery than single MNRs due to their collective effects, such as quorum sensing ability, higher imaging contrast, better motion controllability, bigger drug capacity, and so on (Jin and Zhang, 2021). Fifth, hierarchical multistage targeting strategies to deliver drugs can increase drug delivery accuracy and aggregation at the targeting sites. Sixth, real-time tracking and drug-delivery monitoring may facilitate the autonomous navigation and therapeutic performance of MNRs *in vivo*. For instance, the acoustic report gene has been proven effective in the gastrointestinal tract and cancer models and can provide an image with better resolution than fluorescent protein imaging (Bourdeau et al., 2018). Last but not least, besides the circulatory system, extra attention should be paid to the “motile-targeting” drug delivery of MNRs in less-harsh environments like the urinary, digestive, and reproductive systems, which may expedite the clinical translation of the MNRs.

In summary, recent breakthroughs have been made in the “motile-targeting” drug delivery platforms based on MNRs, with which they can deliver drugs in biofluids with strong thrust, high biocompatibility, and easy navigation. In contrast to traditional TDDSs, they have shown great potential to improve targeting efficiency and reduce side effects. Among the developed MNRs, magnetically-powered and biohybrid MNRs are the most promising candidates to execute drug delivery safely and effectively *in vivo*. Even though there are various challenges, we can foresee and expect clinical trials in the near future, and the great vision of the MNRs operating in human bodies for tumor treatment will finally become reality with continued innovations in design strategies, biointerfacing techniques, swarming control, real-time *in-vivo* imaging, and novel targeting strategies.
